# Riverine bacterial communities are more shaped by species sorting in intensive urban and agricultural watersheds

**DOI:** 10.3389/fmicb.2024.1463549

**Published:** 2024-11-21

**Authors:** Yuanyang She, Peng Wang, Jiawei Wen, Mingjun Ding, Hua Zhang, Minghua Nie, Gaoxiang Huang

**Affiliations:** ^1^School of Geography and Environment, Jiangxi Normal University, Nanchang, China; ^2^Key Laboratory of Poyang Lake Wetland and Watershed Research, Ministry of Education, Jiangxi Normal University, Nanchang, China; ^3^School of History Culture and Tourism, Longnan Normal University, Longnan, China

**Keywords:** landscape pattern, bacterial community, spatial scale, assembly mechanisms, watershed

## Abstract

Bacterial communities play a crucial role in maintaining the stability of river ecosystems and driving biogeochemical cycling, exhibiting high sensitivity to environmental change. However, understanding the spatial scale effects and assembly mechanisms of riverine bacterial communities under distinct anthropogenic disturbances remains a challenge. Here, we investigated bacterial communities across three distinct watersheds [i.e., intensive urban (UW), intensive agricultural (AW), and natural (NW)] in both dry and wet seasons. We explored biogeographic patterns of bacterial communities and the influence of landscape patterns at multi-spatial scales and water chemistry on bacterial communities. Results showed that *α* diversity was significantly lower in UW and AW compared to NW, particularly in the dry season. A gradient of *β* diversity with NW > UW > AW was observed across both seasons (*p* < 0.05). Pseudomonadota, Bacteroidota, and Actinobacteriota were the most abundant phyla across all watersheds, with specific taxa enriched in each watershed (i.e., the class *Actinobacteria* was significant enrichment in UW and AW, and *Clostridia* in NW). The influence of landscape patterns on bacterial communities was significantly lower in human-disturbed watersheds, particularly in UW, where this influence also varied slightly from near riparian buffers to sub-watershed. Homogeneous selection and drift jointly dominated the bacterial community assembly across all watersheds, with homogeneous selection exhibiting a greater influence in UW and AW. Landscape patterns explained less variance in bacterial communities in UW and AW than in NW, and more variance was explained by water chemistry (particularly in UW). These suggest that the stronger influence of species sorting in UW and AW was driven by more allochthonous inputs of water chemistry (greater environmental stress). These findings provide a theoretical foundation for a deeper understanding of riverine bacterial community structure, spatial scale effects, and ecological management under different anthropogenic activities.

## 1 Introduction

Rivers, serving as primary water “sources” for industrial-agricultural production and “sinks” for nutrients and organic matter, play pivotal roles in economic and social development, public health, and environmental protection (Akasaka et al., 2010). Within these ecosystems, bacterial communities play a fundamental role in driving biogeochemical cycles, energy flow, and ecological functioning (Newton et al., 2011). Riverine bacterial communities are highly sensitive to environmental change and are shaped by a complex interplay of natural and anthropogenic factors (Staley et al., 2013; Birrer et al., 2021). The assembly of bacterial communities is influenced by local environment (local factors) and larger spatial regional environment (regional factors) (Lindström and Langenheder, 2012). Allochthonous inputs, nutrient levels (e.g., N, P, C), heavy metals (e.g., Fe, Pb, Cr), and toxic substances can exert strong selective pressures on these bacterial communities (Ajani et al., 2023; Mohapatra et al., 2023). However, stochastic processes such as dispersal limitation, spatial factors, and ecological drift also contribute significantly to shaping bacterial community structure (Wang et al., 2019; Yi et al., 2022; Zhang T. et al., 2022).

The effects of landscape patterns on riverine ecosystems exhibit discernible differences in different anthropogenic activities (Fasching et al., 2020; Zhang Y. et al., 2022). Landscape patterns include landscape compositions (i.e., the proportion of landscape types) and landscape configurations (i.e., the spatial distribution of landscape types) (Xu et al., 2021). With the rapid development of society, anthropogenic activities have significantly changed landscape patterns (urban and agricultural intensification) (Mouri et al., 2011). Intensive urban watershed is often characterized by stronger anthropogenic activities, accompanied by significant substantial industrial production, residential sewage discharge, exogenous bacterial input, and increased impervious surfaces (Samadi Todar et al., 2021; Zhang L. et al., 2021; Murphy et al., 2024). Intensive agricultural watershed often has more agricultural activities, leading to the introduction of various chemical substances (i.e., fertilizers and pesticides) into rivers during the agricultural production process (Allan, 2004; Liu et al., 2024). Intensive urban or agricultural land use leads to an imbalance in the proportion of “source” and “sink” landscapes and changes in spatial configuration, ultimately resulting in the input difference of exogenous bacteria or water chemistry (Germer et al., 2010; Hobbie et al., 2017). Water chemistry acts as a key driver of species sorting, affecting bacterial growth and competition (Guéguen et al., 2004; Gupta et al., 2023; Menéndez-Serra et al., 2023). For example, nitrogen, carbon, and organic matter in river can provide nutrients for microbial growth, while harmful substances such as heavy metals exert stress on microbial growth (Zhang J. et al., 2021, 2023; Shu et al., 2023). Additionally, water chemistry may also influence mass effects via the collective input of allochthonous bacteria (García-Armisen et al., 2014; Staley et al., 2014; Wang et al., 2019). However, the differences in the assembly mechanisms of riverine bacterial communities in intensive urban and agricultural watersheds remain poorly understood (Wang et al., 2019; Sanfilippo et al., 2021; Wu et al., 2022).

Landscape patterns exert their influence on riverine ecosystems across a range of spatial scales, with impacts observed at both local and distant reaches (Paul and Meyer, 2001). Previous studies have examined the influence of near-distance buffer zones and sub-watershed on riverine ecology, and explored whether there are patterns of distance decay across different buffer zones (Zhang et al., 2019; Wu et al., 2021). There is still no consensus on the critical scale of landscape pattern management (Laudon and Sponseller, 2018). For example, some studies emphasizing the importance of the sub-watershed scale (Wang et al., 2023; Xu et al., 2023), and others highlighting the role of riparian buffer zones (Wu and Lu, 2021; Shu et al., 2022). Whether this discrepancy arises due to inherent differences in landscape patterns in different anthropogenically disturbed watersheds remains unclear (Gao et al., 2022; Wu et al., 2022). Most of these studies only analyzed the spatial scale in a single watershed, and the conclusions are less comparable due to differences in climatic environment, watershed size, and anthropogenic disturbances (Liu et al., 2021; Zhu et al., 2022). Further comparative research is needed to elucidate potential distinctions in the spatial effects of landscape patterns on bacterial community structure within watersheds subject to different anthropogenic pressures.

**Figure 1:**
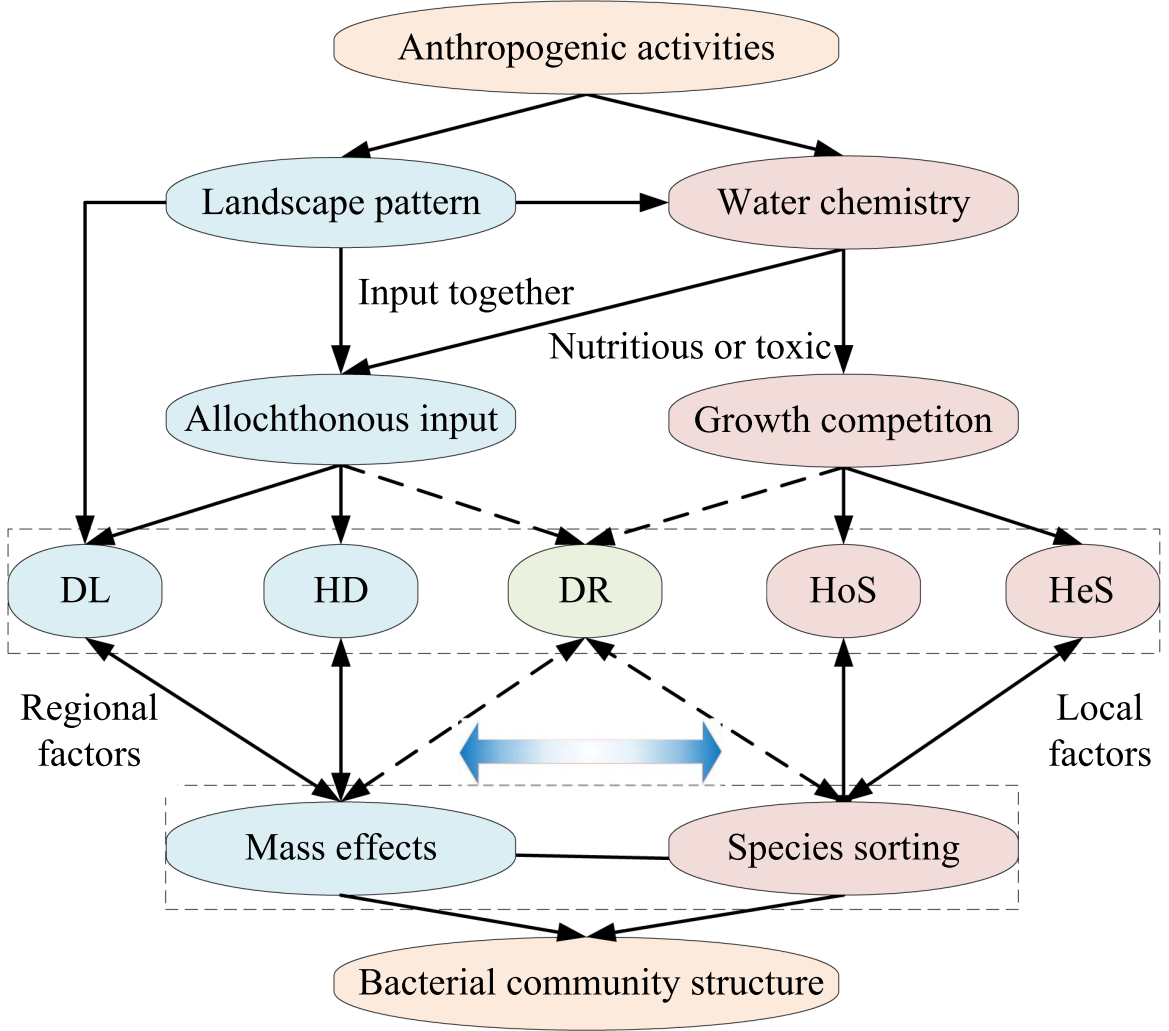
Schematic representation of the influence of landscape pattern and water chemistry on the bacterial community in river water disturbed by different anthropogenic activities. DL, dispersal limitation; HD, homogenizing dispersal; DR, drift; HoS, homogeneous selection; HeS, heterogeneous selection. Gradient blue arrow indicate mechanism-driven strength (Darker color means stronger). Dotted arrows indicate weaker relationships.

This study employed a comparative analysis using high-throughput sequencing to investigate bacterial communities in three intensive watersheds characterized by distinct anthropogenic dominant disturbances [urban, agricultural, and natural (forest)] within a subtropical monsoon zone. Homogeneous selection, heterogeneous selection, and species sorting are mainly effects of the local environment, while homogenizing dispersal, dispersal limitation, and mass effects are mainly effect of the larger spatial regional (Leibold et al., 2004; Stegen et al., 2013; Zhou and Ning, 2017). So, we hypothesized that homogeneous selection and heterogeneous selection are analogous to species sorting, and homogenizing dispersal and dispersal limitation are analogous to mass effects. To disentangle these processes, we considered the unique explanatory ratio of landscape patterns as indicative of mass effects, and the unique explanatory ratio of water chemistry as indicative of species sorting (Figure 1). Our objectives of this study are: (i) to identify the spatial scale effects on riverine bacterial communities under different anthropogenic disturbances, and (ii) to elucidate the distinctions in bacterial community assembly processes and their underlying mechanisms within watersheds experiencing varying anthropogenic pressures.

## 2 Materials and methods

### 2.1 Study area

The study was conducted within the Yuan River watershed, a secondary tributary of the Yangtze River located in the Poyang Lake basin, Jiangxi Province, China (Supplementary Figure S1). Three different watersheds were selected for comparison: the Nanmiao watershed (NMH), characterized by its dominant forest cover (77.74%) and serving as a natural watershed (NW); the Meng watershed (MH), with a predominance of cropland (53.29%) representing an intensive agricultural watershed (AW); and the Kongmu watershed (KMJ) with 8.94% built-up area, passing through the industrial city of Xinyu, and thus experiencing significant influence from urban life and production, constituting an urban watershed (UW) (Supplementary Figure S2; Supplementary Tables S1, S2). The study area belongs to the subtropical monsoon humid climate region, with an average annual temperature of 17.5°C and annual average precipitation of approximately 1,583 mm, primarily concentrated from April to August. The topography of the region is dominated by hills and mountains, resulting in pronounced river catchment effects.

### 2.2 Sample collection and processing

A total of 25 sampling sites were established across the three watersheds, with nine sites in the UW and eight sites each in the NW and AW (Supplementary Figure S1). Water samples were collected at an approximate depth of 50 cm at 25 sites located in the center of the river channel. Each sampling was conducted after a three-day period with no significant precipitation events. In January (dry season) and July (wet season) of 2022, 50 surface water samples were collected using 1 L polyethylene bottles and transported to the laboratory for analysis transported to the laboratory for analysis at the low temperature of 0~4°C. For bacterial community analysis, water samples were filtered through 0.22 μm cellulose acetate membranes (diameter 50 mm; Xingya, China) on the same day, and the filter membranes were stored at −80°C before high-throughput sequencing conducted by Shanghai Meiji Biological Company, China. Electrical conductivity (EC), water temperature (WT), pH, and dissolved oxygen (DO) were measured *in situ* using an HI 98360 probe (Hanna Instruments Ltd., Italy) that was calibrated before each measurement. Water samples for water chemistry measurement were filtered through a 0.45 μm pore size cellulose acetate membrane, sealed in a sampling bottle, and stored at 0~4°C. Dissolved organic carbon (DOC) was measured using a TOC analyzer (Shimadzu TOC-L CPH, Japan), while chlorine ions (Cl^−^) and sulfate ions (SO_4_^2−^) were determined by ion chromatography system (ICS-600). The nitrate and ammonia nitrogen (NO_3_^−^-N, NH_4_^+^-N) and total phosphorus (TP), were quantified using an automatic discontinuous analyzer (Smartchem 200 Brookfield, United States) with the Environmental Monitoring method standard of the Ministry of Ecology and Environment, PRC. The concentrations of chromium (Cr), iron (Fe), manganese (Mn), copper (Cu), cadmium (Cd), and lead (Pb) were measured by inductively coupled plasma-mass spectrometry (ICP-MS, Thermo X series II, USA). The accuracy and precision of the methods and results were checked by using the certified Standard Reference Materials (SRM-1640 and SRM-1643e of National Institute of Scientific and Technology, United States).

### 2.3 Landscape patterns and buffer zone division

Digital elevation model (DEM) data with a 12.5 m resolution, was obtained from the NASA Earth Science Data website.^1^ Utilizing the ArcSWAT module within ArcGIS, sub-watershed and riparian buffers at 1,000 m, 500 m, 300 m, and 100 m scale were extracted based on rivers and sampling points (Supplementary Figure S1). Land use data for the year 2020 were obtained from the World Cover dataset published by the European Aviation Agency, featuring a grid resolution of 10 m.^2^ This study based on prior research (Chen et al., 2018; Zhou et al., 2020; Sanfilippo et al., 2021) that trees and shrubs were combined as “Forest,” while grasslands and sparse vegetation were combined as “Grassland.” The categories of agricultural and built-up were maintained. In aggregate, these categories encompass in excess of 97% of the total area within each watershed. The remaining categories account for a minor proportion of the total, and thus were not considered in further analysis (Supplementary Tables S1, S2; Supplementary Figure S2). Landscape configuration indices, specifically patch density (PD) and mean Euclidean nearest neighbor distance (ENN_MN), were calculated at the landscape level using Fragstats 4.2 software to quantify landscape fragmentation and connectivity (Supplementary Tables S3, S4).

### 2.4 DNA extraction, 16S rRNA gene amplification and sequencing

DNA was extracted from 0.5 g of well-mixed samples using the FastDNA SPIN Kit for soil (MP BIO, Qbiogene, United States) according to the manufacturer’s instructions. The bacterial V3–V4 hypervariable regions of 16S rRNA genes were amplified using a forward primer of: 338F (5′ACTCCTACGGGAGGCAGCA-3′) and a reverse primer of: 806R (5′-GGACTACHVGGGTWTCTAAT-3′) (Castrillo et al., 2017). The V3–V4 hypervariable region has been targeted via the MiSeq platform (which can produce single-end reads of 350 bp), which can allow for more accurate and cost-effective characterizations of microbiome samples (Caporaso et al., 2012; Fouhy et al., 2016). The PCR reaction was carried out in triplicate at 94°C for 5 min with an initial denaturation, followed by denaturation at 94°C for 30 s, annealing at 52°C for 30 s, extension at 72°C for 30 cycles, and a final extension step at 72°C for 10 min. The PCR products were purified, quantified, and combined for library preparation using the NEBNext^®^ Ultra^™^ DNA Library Prep Kit according to the manufacturer’s recommendations. During this step, three negative controls were used to verify no contamination during DNA extraction and PCR amplification. All PCR products were sequenced using the Illumina MiSeq platform by Shanghai Meiji Biological Company, China.

### 2.5 Data processing and analyses

Raw sequence data were processed using the QIIME2^3^ (Bolyen et al., 2019), followed by denoising and filtering using DADA2 (Callahan et al., 2016). Amplicon sequence variants (ASVs) were assigned based on 100% sequence similarity to the same sample sequence. *α* and *β* diversity metrics were calculated using the “vegan” package in R. Geographical distances between sampling sites were calculated based on latitude and longitude coordinates using the ‘geosphere’ package. Data normality was assessed using the Shapiro–Wilk test, and non-parametric Kruskal-Wallis tests were employed for data that did not conform to a normal distribution (*p* < 0.05). The “Bioenv” function (Clarke and Ainsworth, 1993) was used to select a subset of landscape patterns and water chemistry for further analysis. To reduce distribution heteroscedasticity, all environmental variables (except pH) were natural logarithm (ln) transformed before statistical analyses.

Principal coordinates analysis (PCoA) based on Bray-Curtis distances was used to visualize differences in bacterial community structure, with significance assessed using ANOSIM tests. To relate environmental variables to specific ASVs, we performed variation partitioning (“varpart” function in R) followed by canonical correspondence analysis (CCA) using the “cca” and “anova.cca” functions in the “vegan” R package. Variance partitioning analysis (VPA) (Borcard et al., 1992), as an effective method, was further used to quantify the relative contributions of landscape patterns and water chemistry to bacterial community variations. The phylogenetic normalized stochasticity ratio (pNST) was calculated using the “NST” Package. Null model analysis was carried out using the framework of Ning et al. (2020) to classify community pairs into underlying driving forces of deterministic [e.g., homogeneous selection (HoS), heterogeneous selection (HeS)] and stochastic [e.g., homogenizing dispersal (HD), dispersal limitation (DL), and “drift” (DR)]. Null model analyzed the proportion of each construction process using the big function (limit size = 24) in the iCAMP package in R (Ning et al., 2020). Linear discriminant analysis coupled with effect size (LEfSe), LDA score (log 10), was used for identifying the taxa differences among various aggregates at the genus levels (Segata et al., 2011) and visualized at ternary plots. All statistical analyses were performed in R software (v. 4.4.1).

## 3 Results

### 3.1 Diversity and composition of the bacterial community

**Figure 2:**
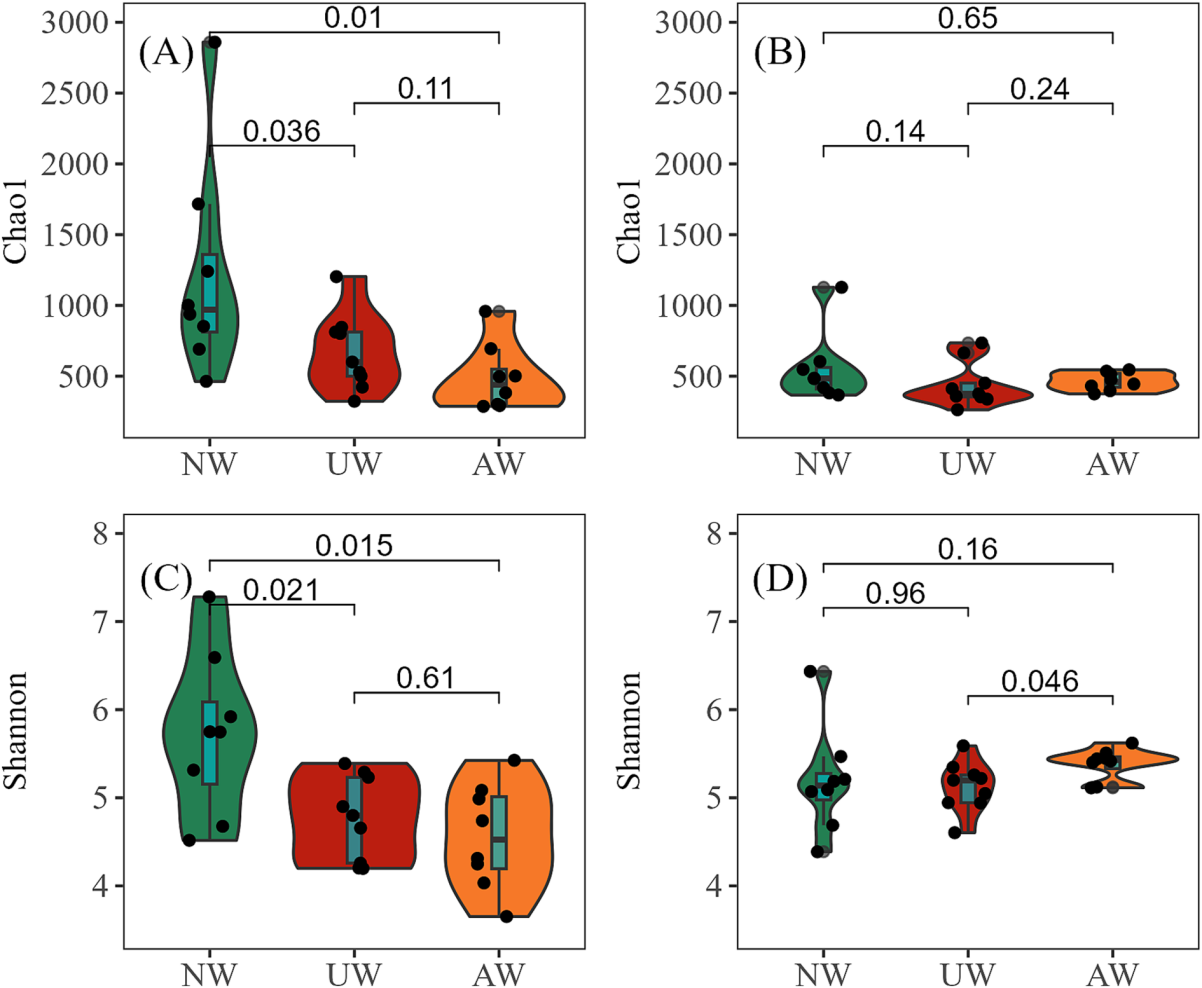
*α* diversity of bacterial communities in different watersheds (Wilcox. test). (A,B) Represent Chao1 richness in the dry and wet seasons, respectively; (C,D) represent Shannon diversity in the dry and wet seasons, respectively.

In both dry and wet seasons, a total of 12,932 ASVs were assigned to 2,921 species, 1,396 genera, 644 families, 367 orders, 151 classes, and 52 phyla. In the dry season, *α* diversity, as measured by the Chao1 richness and Shannon diversity indices, was highest at site N1 (2859.40/7.28) and lowest at sites M7 (Chao1: 301.57) and M6 (Shannon: 3.65) (Supplementary Figure S3). A clear gradient in *α* diversity was observed among the three watersheds, with NW exhibiting the highest values, followed by UW and then AW, with significant differences among the three watersheds (*p* < 0.05). In contrast, in the wet season, no significant differences in *α* diversity were observed among the watersheds, except for a higher Shannon diversity in AW compared to UW (*p* < 0.05) (Figure 2). Similarly, the highest values of Chao1 richness index and Shannon diversity index (1127.91/6.43) were found at site N1, whereas the lowest Chao1 richness index (263.21) and Shannon diversity index (4.38) were found at sites K3 and N5, respectively (Supplementary Figure S3). The Chao1 richness index of the UW and AW had significant differences between the two seasons (*p* < 0.05). Shannon diversity showed no significant variation across any of the watersheds (*p* > 0.05) (Supplementary Figure S4). Spatial patterns along the river continuum were also evident in the dry season, with significantly higher Chao1 richness and Shannon diversity in upstream reaches compared to downstream reaches (*p* < 0.05). However, these spatial patterns disappeared in the wet season (Supplementary Figure S5).

**Figure 3:**
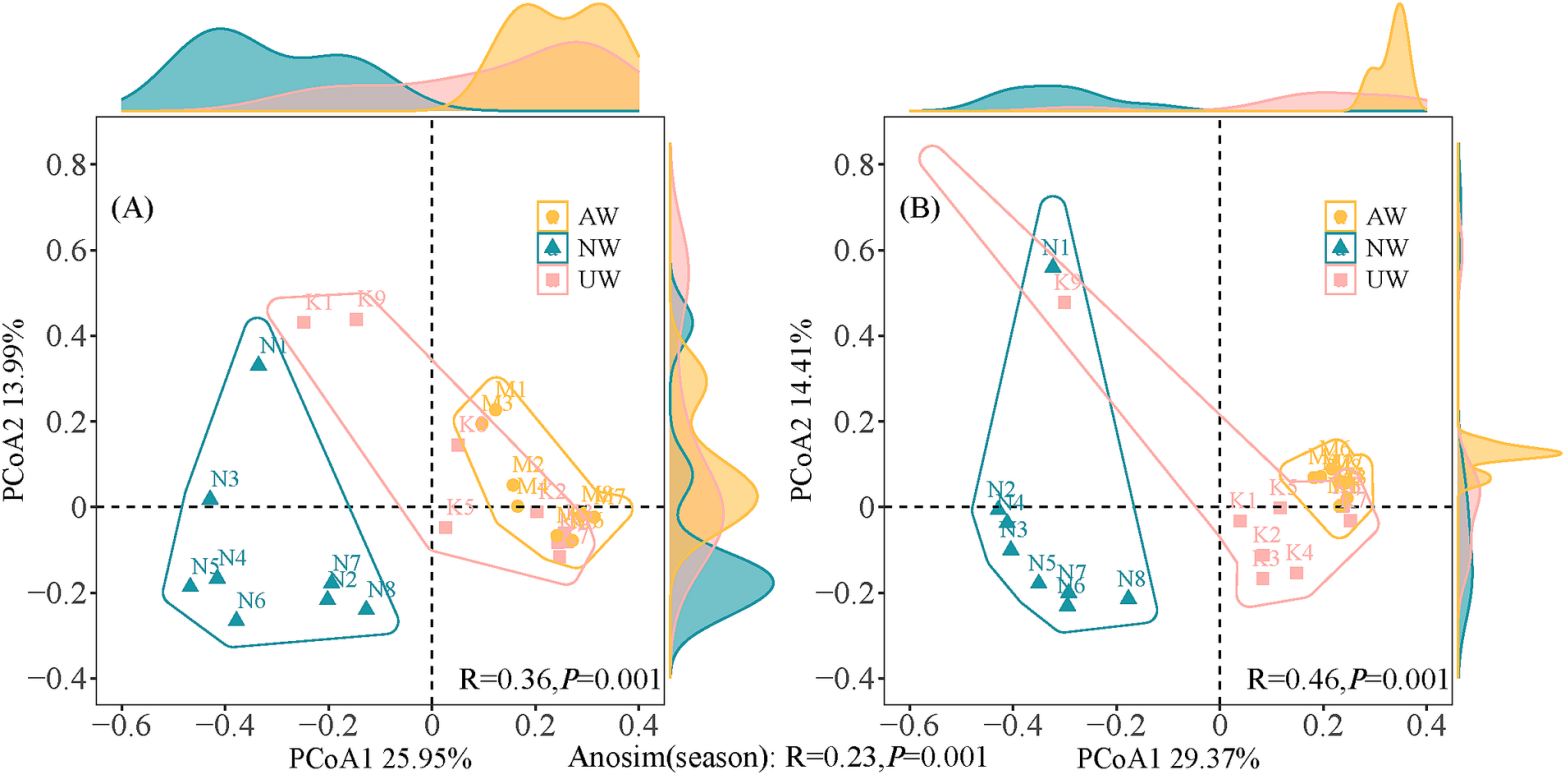
Principal co-ordinates analysis (PCoA) and analysis of similarity tests (ANOSIM) based on Bray-Curtis distances in the dry (A) and wet (B) seasons.

To better understand how the bacterial communities varied across space and time, we used Principal Coordinate Analysis (PCoA) and ANOSIM tests based on the relative abundance of bacteria (Figure 3). The PCoA plot separated the samples into two distinct groups based on season, a finding confirmed by the ANOSIM test (*R* = 0.23, *p* < 0.01). This indicates a significant difference in bacterial community composition between the dry and wet seasons. Furthermore, the *β* diversity analysis revealed significant differences among the three watersheds (AW, NW, UW) in both seasons, with even stronger separation observed in the PCoA plots and higher ANOSIM values (dry season: *R* = 0.36, *p* < 0.01; wet season: *R* = 0.46, *p* < 0.01). The distribution of sample points in the PCoA plots offered further insights. In both seasons, NW and UW samples were more scattered, suggesting greater variation in bacterial communities within these watersheds. In contrast, AW samples were more tightly clustered, particularly in the wet season, indicating greater similarity among their bacterial communities. Notably, sites N1 and K9 consistently stood out from the others in both seasons, indicating that they were significantly different from the other sites.

**Figure 4:**
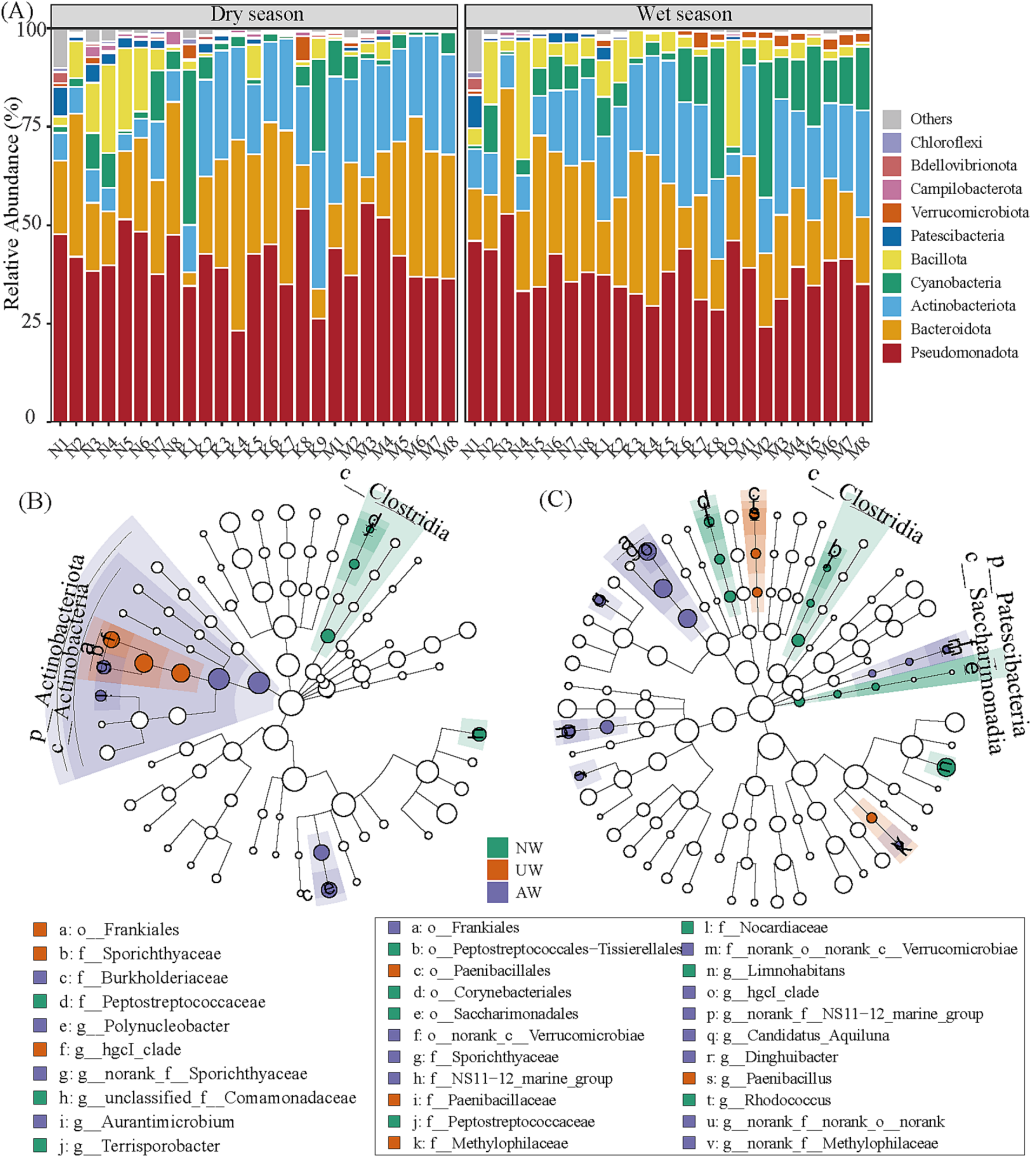
The relative abundances of dominant phyla in the bacterial community (A). Linear discriminant analysis (LDA) effect size (LEfSe) (LDA > 3.5, *p* < 0.05) to identify biomarkers in different rivers, (B,C) represent the dry season and wet seasons, respectively. The taxa with significant differences in UW, AW, and NW application are represented by red, purple, and green dots, respectively, and the taxa with no significant differences are not shown in the dendrogram. The points from the center to the outer sphere represent the level of phylum (p_), class (c_), order (o_), family (f_), and genus (g_).

In both dry and wet seasons, Pseudomonadota was the most abundant phylum (41.46 ± 7.74%), followed by Bacteroidota (23.79 ± 11.31%), Actinobacteriota (18.73 ± 9.05%), Cyanobacteria (6.20 ± 8.41%), Bacillota (1.43 ± 1.70%), Patescibacteria (0.83 ± 1.35%), and Verrucomicrobiota (0.67 ± 0.86%) (Figure 4A). At the genus level, *Limnohibatans* had the highest relative abundance (9.19 ± 6.80%), followed by *Flavobacterium* (6.65 ± 7.67%), *Pseudarcicella* (5.42 ± 5.79%), *hgcI_clade* (5.20 ± 3.78%), *unclassified_f__Comamonadaceae* (5.01 ± 3.38%) (Supplementary Figure S6). The number of ASVs in each watershed was higher in the dry season than in the wet season. The order of the unique ASVs was NW (5,136/2167) > UW (1922/1156) > AW (953/599) in the dry/wet season. The total number of shared ASVs followed the order NW∩UW (935) > AW∩UW (806) > NW∩UW (658) in the dry season and AW∩UW (670) > NW∩UW (525) > NW∩UW (428) in the wet season (Supplementary Figure S7).

To pinpoint key bacterial groups characteristic of each watershed, we employed LEfSe analysis, which identified 12 and 8 biomarkers in the dry and wet seasons, respectively (Figures 4B,C; Supplementary Table S7). In the dry season, Actinobacteriota, especially the class *Actinobacteria* (e.g., *Frankiales* and *Micrococcales*), thrived in both UW and AW, while Firmicutes (e.g., *Clostridia* and *Bacilli*) were enriched in NW. The wet season witnessed a shift, with Patescibacteria, *Actinobacteria* (*Frankiales* and *Micrococcales*), and specific orders of Bacteroidota becoming signatures of NW and AW. Meanwhile, UW was characterized by the orders Paenibacillales (class *Bacilli*) and *Burkholderiales* (class *Gammaproteobacteria*). These findings suggest the potential ecological roles of these bacterial groups within each watershed and across seasons.

**Figure 5:**
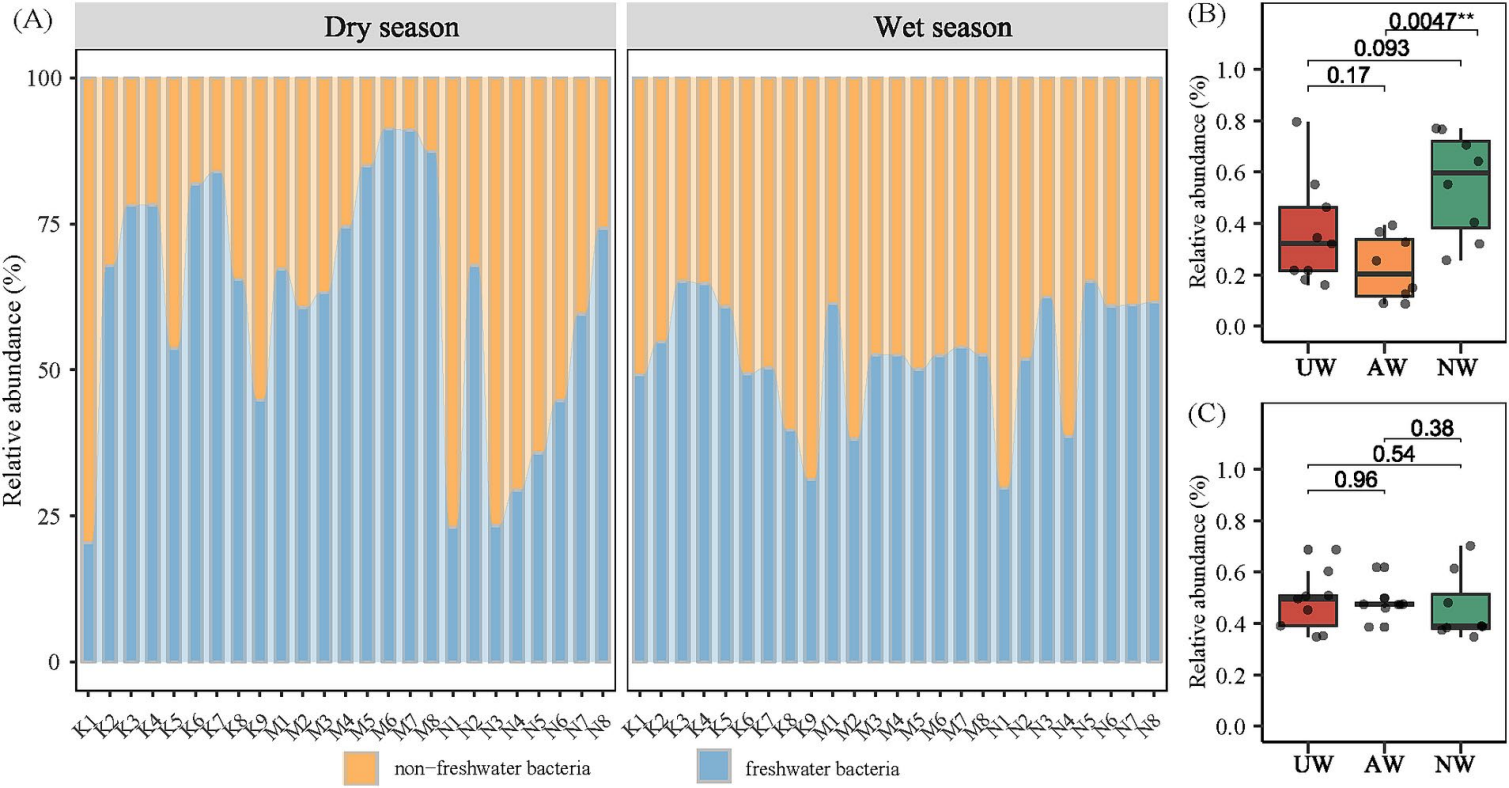
Proportions of sequence reads assigned to the typical freshwater lineages and non-freshwater bacteria according to the definition in the Newton freshwater database (A). Difference comparison of non-freshwater bacterial in different watersheds in the dry season (B) and the wet season (C) using Kruskal test.

By referencing the freshwater-specific 16S rRNA gene database (Newton et al., 2011), we classified all 12,932 identified bacterial species (ASVs) as either typical freshwater or non-freshwater bacteria. Our analysis revealed a noteworthy presence of non-freshwater bacteria, with their relative abundance ranging from 8.73 to 79.57% (average: 37.88%) in the dry season and 34.81 to 70.23% (average: 47.57%) in the wet season. This difference between seasons was statistically significant, indicating a higher proportion of non-freshwater bacteria in the wet season (Figure 5A). Among the watersheds, NW exhibited the highest relative abundance of non-freshwater bacteria in the dry season, significantly exceeding that of AW (*p* < 0.01) (Figure 5B). Previous studies have suggested non-freshwater bacteria primarily originate from external sources, such as the surrounding terrestrial ecosystem (Wang et al., 2019).

### 3.2 Spatial scale effects on bacterial community structure

“Bioenv” analysis and the Mantel test were used to investigate the optimal spatial scale at which landscape patterns influence bacterial communities across three watersheds (Table 1). Notably, the influence of landscape patterns on bacterial community structure generally decreased from NW to AW to UW. In UW, there were slight differences in landscape patterns on bacterial communities at different spatial scales in the dry and wet seasons. For instance, the highest correlation (0.63) was observed at the 100 m riparian buffer zone in UW in the dry season, followed by the 500 m and sub-watershed (0.53). The Grassland, the Built-up, and the Built-up & ENN_MN were the most significant variables influencing the bacterial community in the 100 m riparian buffer zone, 500 m riparian buffer zone, and sub-watershed, respectively. Similar trends persisted in the wet season, with Built-up & PD and Built-up & ENN_MN being most influential at the 500 m and sub-watershed scales, respectively. In AW, correlations were slightly weaker at the sub-watershed scale than at the riparian buffer, particularly in the dry season. The 500 m riparian buffer exhibited the highest correlation (0.84), with Cropland and Grassland as the main drivers of bacterial community structure. Interestingly, in the NW and AW in the wet season, the effect on bacterial communities did not differ significantly between the riparian buffer and sub-watershed scales. Grassland, Built-up, and ENN_MN were identified as the primary factors shaping the bacterial community in the NW. The comprehensive analysis showed that the landscape patterns at the 500 m riparian buffer can better explain the variations of bacterial communities across three watersheds. Consequently, landscape pattern variables at this scale were selected for subsequent analyses.

**Table 1:** The correlation and optimal variables of landscape patterns on the bacterial community at different spatial scales based on Bioenv.

Watershed	Scale	Dry season		Wet season	
		Optimal variables	Correlation	Optimal variables	Correlation
UW	100 m	Gr	0.63^**^	Gr	0.44
	300 m	PD	0.35	PD	0.33
	500 m	Bu	0.53^*^	Bu, PD	0.48^*^
	1,000 m	Cr, Bu	0.47	Cr, Bu, PD	0.39
	Sub-watershed	Bu, EN	0.53^*^	Bu, EN	0.49^*^
	Water chemistry	Cl^−^, WT, DOC, NH_4_^+^-N, Cu, Fe	0.78^*^	DO, WT, NH_4_^+^-N, Mn, Pb, Fe	0.87^**^
AW	100 m	Gr, Cr, Bu	0.79^**^	Fo, Gr, Cr	0.87^**^
	300 m	Gr, Cr, Bu	0.81^**^	Gr, Bu, PD	0.88^**^
	500 m	Gr, Cr	0.84^*^	Gr, Bu	0.81^**^
	1,000 m	Gr, Cr	0.79^**^	Gr, PD, EN	0.86^**^
	Sub-watershed	Gr, Bu PD	0.68^**^	Gr, PD	0.83^**^
	Water chemistry	SO_4_^2−^, EC, DOC, TP, NH_4_^+^-N, Cu	0.86^**^	Cl-, SO_4_^2−^, EC, WT, NO_3_^−^-N, Fe	0.85^**^
NW	100 m	Gr, Cr, Bu	0.89^**^	Gr, Cr, Bu, EN	0.94^**^
	300 m	Gr, Bu, PD, EN	0.90^**^	Cr, PD, EN	0.95^**^
	500 m	Gr, Cr	0.89^**^	Cr, Bu, EN	0.90^**^
	1,000 m	Gr, Cr	0.90^**^	Cr	0.90^*^
	Sub-watershed	Fo, Gr, Bu, PD, EN	0.90^**^	Gr, Cr	0.89^**^
	Water chemistry	Cl^−^, SO_4_^2−^, pH, EC Cu, Fe	0.95^**^	SO_4_^2−^, WT, NH_4_^+^-N, Mn, Pb, Fe	0.89^**^

Gr, Grassland; Bu, Built-up; Cr, Cropland; Fo, Forest; PD, Patch density; and EN, ENN_MN. ^*^Correlation is significant at the 0.05 level; ^**^significant at the 0.01 level using Mantel test.

### 3.3 Effects of landscape patterns and water chemistry on bacterial community structure

**Figure 6:**
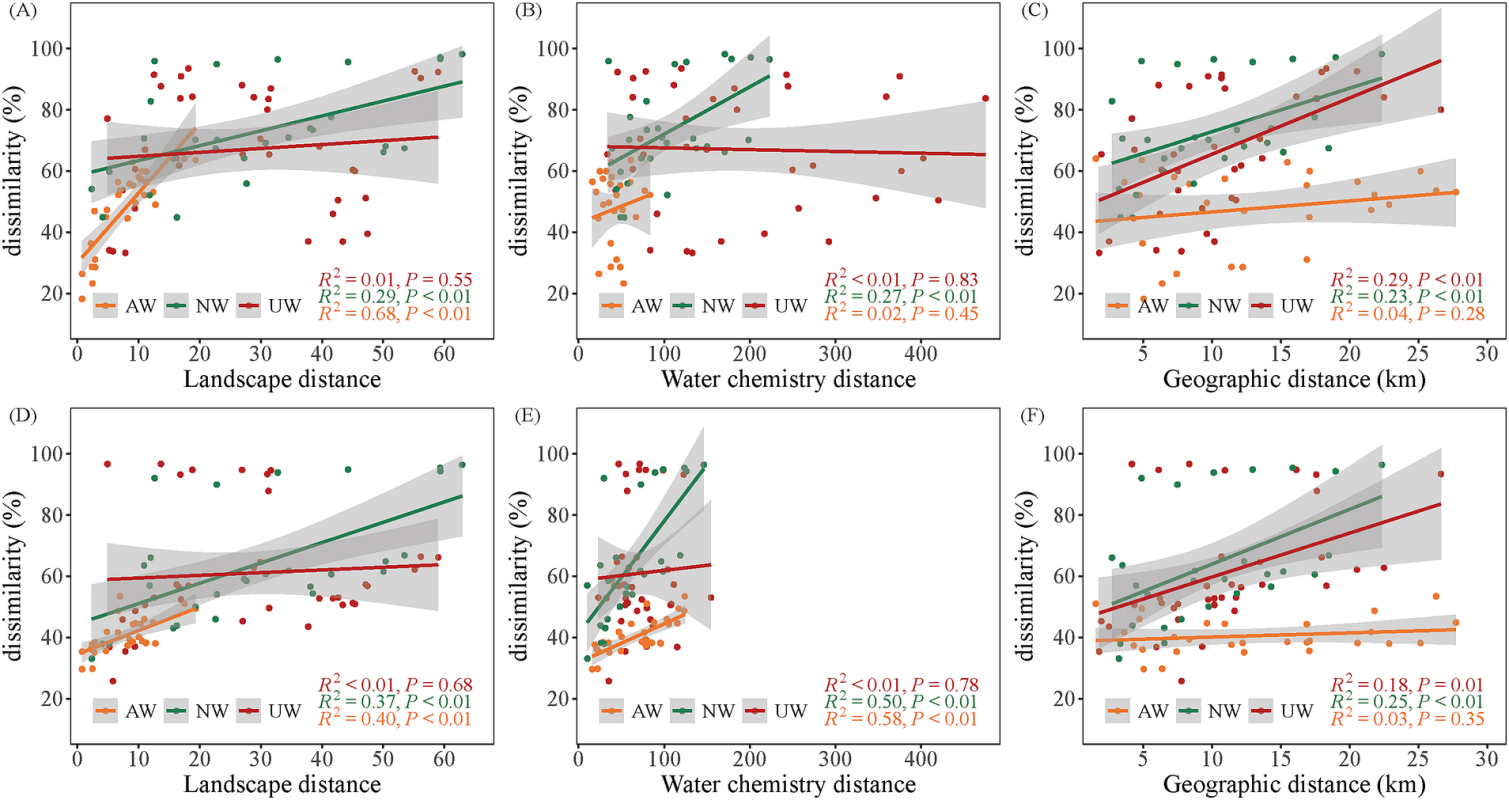
Relationships between *β* diversity (Bray–Curtis dissimilarity) and landscape patterns (Euclidean distance), water chemistry (Euclidean distance), and geographic distance. (A–C) Show linear regression relationships of landscape patterns, water chemistry and geographic distances with *β* diversity in the dry seasons, respectively. (D–F) Show linear regression relationships of landscape patterns, water chemistry and geographic distances with *β* diversity in the wet seasons, respectively.

To evaluate the influence of landscape patterns and water chemistry on bacterial community structure, we employed linear regression models and redundancy analyses. Our results revealed distinct patterns across the three watersheds. The Euclidean distances of landscape patterns were more similar in the AW than in the NW and UW, indicating that landscape pattern structures in the AW were more similar. Moreover, a positive correlation between landscape pattern distance and Bray–Curtis dissimilarity was observed in both AW (*R*^2^ = 0.67/0.38) and NW (*R*^2^ = 0.26/0.35) in the dry/wet seasons, respectively (*p* < 0.05) (Figures 6A,D). A gradient of water chemistry distances was observed with UW > NW > AW in both seasons. Notably, these distances decreased significantly in the dry season compared to the wet season, particularly in the UW. In the dry season, only the NW showed a significant positive correlation between water chemistry distance and Bray–Curtis dissimilarity (*R*^2^ = 0.24, *p* < 0.05). However, in the wet season, both AW (*R*^2^ = 0.56) and NW (*R*^2^ = 0.48) displayed significant positive correlations (*p* < 0.05) (Figures 6B,E). Interestingly, the AW did not exhibit significant distance decay patterns in either season (*p* > 0.05) (Figures 6C,F). This further supports the observation of homogeneity in both landscape patterns and water chemistry within the AW, suggesting a more uniform environment compared to the NW and UW.

**Figure 7:**
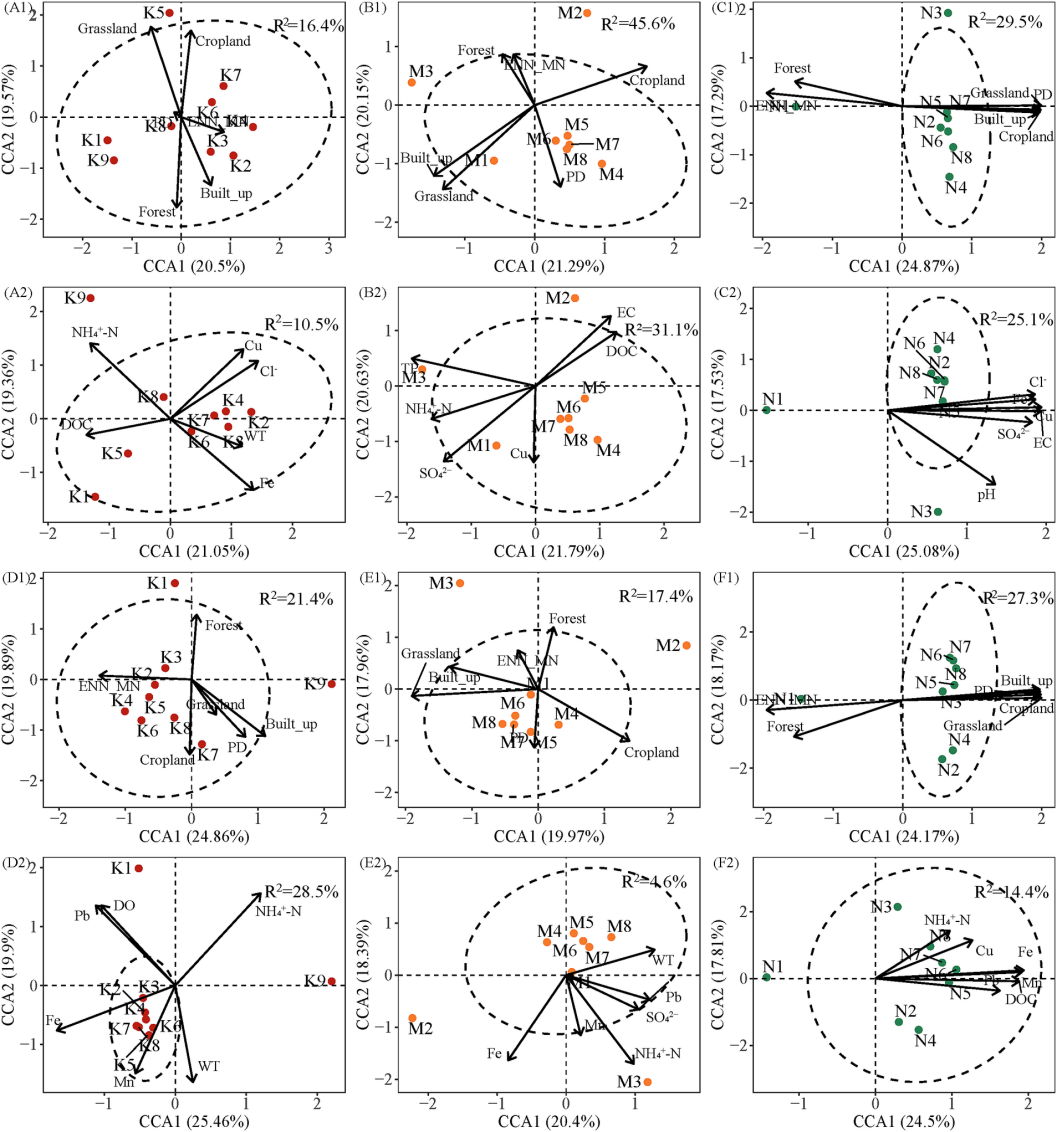
Canonical correspondence analysis (CCA) was performed to determine the most significant landscape patterns and water chemistry variables shaping bacterial communities in the different watersheds. Blue and red arrows indicate landscape patterns and water chemistry variables, respectively. *R*^2^ represents adjustment *R*^2^. (A1–C1) Show the relationship between landscape patterns and bacterial community in the dry season, respectively. (A2–C2) Show the relationship between water chemistry and bacterial community in the dry season, respectively. (D1–F1) Show the relationship between landscape patterns and bacterial community in the wet season, respectively. (D2–F2) Show the relationship between water chemistry and bacterial community in the wet season, respectively. Abbreviations of environmental variables are defined in the “Materials and methods” section.

Canonical Correspondence Analysis (CCA) revealed the influence of both landscape patterns and water chemistry on bacterial community composition across the three watersheds. The first two CCA axes explained 37.93–44.75% of the variance in community composition when considering landscape patterns (Figure 7). The highest explanatory ratio was observed in the UW in the wet season (44.75%), while the lowest was observed in the AW in the same season (37.93%) (Figures 7D1,E1). The first and second ordination axes in CCA together accounted for 38.79 ~ 45.36% of the variance in community composition in the dry and wet seasons with water chemistry, respectively. The highest and lowest explanatory rates were found in the UW (45.36%) and AW (38.79%) in the wet season (Figures 7D2,E2). In the dry season, the bacterial community was the most significant landscape pattern variable shaped by Grassland (*F* = 1.44, *p* < 0.01), Grassland (*F* = 2.16, *p* < 0.01), and Forest (*F* = 1.62, *p* < 0.01) in UW, AW, and NW, respectively. Cl^−^ (*F* = 1.31, *p* < 0.01), SO_4_^2−^ (*F* = 1.70, *p* < 0.01), and Cl^−^ (*F* = 1.69, *p* < 0.01) were the most significant water chemistry variables in UW, AW, and NW. In the wet season, the bacterial community was most significantly the landscape patterns variables shaped by Built-up (*F* = 1.71, *p* < 0.01), Grassland (*F* = 1.47, *p* < 0.01), and Forest (*F* = 1.64, *p* < 0.05) in the UW, AW, and NW. The bacterial community was most significantly the water chemistry variables influenced by DO (*F* = 1.75, *p* < 0.01) and WT (*F* = 1.15, *p* < 0.01) in the UW and AW (Supplementary Table S5). Notably, a greater number of landscape pattern variables exhibited significant correlations with bacterial communities compared to water chemistry variables in both dry and wet seasons. However, it was also observed that there was a high interpretation rate of landscape patterns for water chemistry (Supplementary Figure S8). These results highlight the complex interplay between landscape features and water chemistry in shaping bacterial communities within each watershed.

### 3.4 Assembly mechanisms of bacterial community structure in different watershed

**Figure 8:**
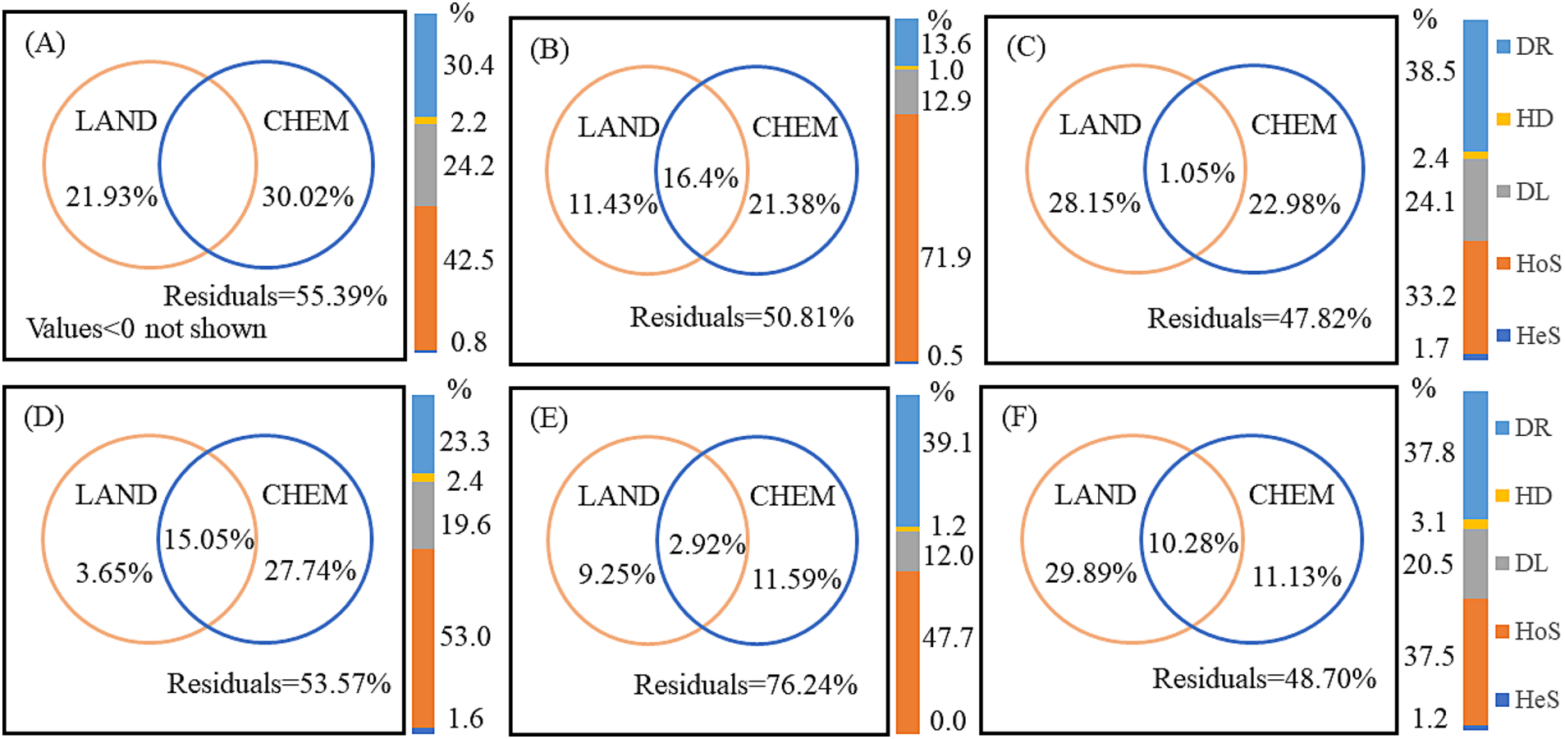
Variation partitioning analysis (VPA) quantified the contribution of landscape pattern (LAND) and water chemistry (CHEM) variables to bacterial community variation across watersheds and seasons. Values less than zero are not shown. Bar diagrams represent the proportion of ecological processes in the bacterial communities. DR, drift; HD, homogenizing dispersal; DL, dispersal limitation; HoS, homogeneous selection; HeS, heterogeneous selection. (A–C) Represent UW, AW, and NW in the dry season, respectively. (D–F) Represent UW, AW, and NW in the wet season, respectively.

VPA was used to quantify the relative contributions of landscape pattern, water chemistry, and their interactions to bacterial community variation (Figure 8). Forward selection models identified the specific landscape pattern and water chemistry variables with significant effects on community structure (Supplementary Table S6). Interestingly, the explanatory ratios of water chemistry on bacterial community variation decreased as increasing anthropogenic activity, with significantly lower contributions in the UW and AW compared to the NW in both dry and wet seasons. This decrease was particularly evident in the dry season, with water chemistry explaining only 11.43 and 3.65% of the variations in AW and UW, respectively. This suggests a decreasing influence of “mass effects” on bacterial communities as human activities increase. Notably, unexplained variation in the AW was highest (76.24%) in the wet season, suggesting the presence of additional influencing factors.

Null model analysis revealed that deterministic processes played a greater role in shaping bacterial communities in the UW and AW compared to the NW, as indicated by the lower pNST values (<0.5) in the former, especially in the AW during the wet season (Supplementary Figure S9). This indicated that deterministic processes and stochastic processes collaborated in shaping the bacterial communities in each watershed, and deterministic process was more pronounced in the UW and AW than in the NW. Among the deterministic processes, HoS emerged as the dominant force in all watersheds, and the order of HoS was AW (71.9%) > UW (42.5%) > NW (33.2%) in the dry season, and UW (53.0%) > AW (47.7%) > NW (37.5%) in the wet season. Stochastic processes also played a role, primarily driven by DL and DR. The influence of DR was significantly reduced in UW (30.4%) and AW (13.6%) compared to NW (38.5%) in the dry season, suggesting that agricultural activities may enhance the role of HoS while reducing the influence of DR. Furthermore, DL was significantly lower in AW compared to UW and NW (Figure 8).

## 4 Discussion

### 4.1 Diversity and composition differences of bacterial community structure

Our study revealed a significant difference in microbial diversity among the three watersheds in the dry season. NW exhibited the highest Chao1 richness and Shannon diversity compared to the AW and UW in the dry season (*p* < 0.05). This aligns with previous research indicating that regions with intensive agriculture and industry often harbor lower microbial diversity (Ibekwe et al., 2016; Li et al., 2020). The microbiota is mainly controlled by local environmental factors, and exhibiting a spatial pattern of greater species diversity in headwater (low environment stress) than downstream (Besemer et al., 2013; Savio et al., 2015). It should be noted that the sites within NW were less influenced by pollutants. This conclusion is further supported by the fact that NW has the highest *α* diversity of all samples (i.e., site N1) (Supplementary Figure S3). More surface water directly flows into rivers through the upper of the impervious layer in UW compared to NW, and the rich microorganisms of the soil beneath the impermeable layer are not easily carried into the river. This might also help explain the low *α* diversity observed in the UW. In AW, large agricultural areas have decreased landscape heterogeneity (Figure 6), leading to a decrease in the diversity of exogenously imported bacterial communities. Furthermore, both AW and UW experience significant anthropogenic activities that introduce pollutants such as antibiotics, sewage, fertilizers, and pesticides. These stressors can negatively impact bacterial communities and contribute to lower diversity (Liu et al., 2023; Zhang et al., 2023; Zou et al., 2023). Interestingly, in the wet season, the differences in *α* diversity among watersheds became less pronounced, with only the Shannon diversity in AW being slightly higher than in UW (*p* < 0.05). This could be due to the higher temperature in the wet season promotes microbial growth (Fang et al., 2023), while increased rainfall leads to higher water levels and flow, potentially homogenizing bacterial communities and reducing diversity differences between watersheds (Chen et al., 2019). However, this is inconsistent with some of the previous conclusions. For instance, previous studies have shown that the *α* diversity index of river bacterial community increased significantly with the increase of human activity intensity (the proportion of built-up increased) (Zhou et al., 2020; Wu et al., 2022). These discrepancies may be due to differences in the level of anthropogenic interference, watershed scale, or the specific microbial communities studied.

Here, Pseudomonadota, Bacteroidota, Actinobacteriota, Cyanobacteria, and Bacillota are dominant phyla in the three watersheds (Figure 4), and the widespread presence of these major bacterial lineages in various riverine habitats has been suggested (Zhao et al., 2021; Wu et al., 2022; Zou et al., 2023). From the difference analysis, C_*Actinobacteria* was significantly enriched in the AW and UW, especially the AW. C_*Actinobacteria* are a highly diverse group of bacteria renowned for their remarkable metabolic versatility. They are responsible for the production of most clinically used antibiotics and a large number of other natural products with medical and agricultural applications (van Bergeijk et al., 2020). An estimated 64% of all naturally occurring antibiotics are derived from Actinobacteriota species (Hutchings et al., 2019). Their capabilities extend beyond antibiotic production, as they exhibit antipathogenic activity, enhance plant biomass production, and induce plant disease resistance. As a result, C_*Actinobacteria* are commonly utilized as biofertilizer inoculants, contributing to sustainable agricultural practices (Olanrewaju and Babalola, 2019; Li et al., 2022). Species sorting facilitates the growth of Actinobacteria that specialize in k-strategies (with slow growth, small size, and less susceptible to predation avoidance) (van Teeseling et al., 2015). C_*Gammaproteobacteria* were also significantly enriched in the AW in the dry season. This group has been identified as the core bacteria with the highest colonization potential in the gut, and was further found to be the best indicator taxon of the response to environmental concentrations of soil pollution. Furthermore, the abundance of C_*Gammaproteobacteria* is closely correlated with the presence of antibiotic-resistance genes (Zhang Q. et al., 2021). The NW watershed, on the other hand, showed significant enrichment of C_*Clostridia* and C_*Bacilli* (O_*Erysipelotrichales*). These bacteria are ubiquitous in nature, commonly found in soil samples and the human intestine. The high abundance of these groups in the NW may be related to the large number of settlements close to the riparian zone, where untreated human and poultry waste may enter the river system.

The bacterial composition showed intriguing seasonal and spatial variations. Notably, C_*Bacteroidia* (O_*Chitinophagales*) and C_*Bacilli* (O_*Paenibacillales*) in the wet season were more abundant in AW and UW, respectively. The presence of C_*Bacteroidia*, often found in human and animal intestines, suggests potential fecal contamination, aligning with previous research (Wéry et al., 2008; Ibekwe et al., 2016). Interestingly, soils with high NO_3_^−^-N (UW and AW) had more C_*Bacteroidia* and a complete loss of Firmicutes (Kandasamy et al., 2021). Thus, Bacteroidetes are detected consistently at all sites in both two seasons, suggesting that they come from anthropogenic inputs (Dubinsky et al., 2012). More runoff can carry such bacteria into the river in pipes or farther villages and towns. These microbes may be active in microbe nutrient interactions. On the other hand, C_*Parcubacteria* (phylum Patescibacteria) were significantly enriched in the NW, especially in the wet season. These bacteria are commonly found in groundwater, sediments, lakes, and other aquifer environments (Proctor et al., 2018; Tian et al., 2020). Their small cell size (~0.3 μm) is likely an adaptation to low-nutrient conditions, facilitating increased nutrient uptake and metabolic rates due to a higher surface-area-to-volume ratio (Savage et al., 2007; Tian et al., 2020).

### 4.2 Spatial scale effects of landscape pattern on bacterial community structure

The results showed that the correlations between the landscape patterns and bacterial community structure showed an obvious decrease in UW compared to NW in both the dry and wet seasons, and a slightly decrease in AW. Prior studies have demonstrated that landuse types within watersheds, such as agricultural and urban landuse are significant drivers of the structure of the aquatic biota (e.g., bacteria, zooplankton, and phytoplankton) (Uchida et al., 2021; Yang et al., 2022; Zhang Y. et al., 2022). Urbanization is the process of anthropogenic transformation of wildlands or agricultural land into built environments where human habitation and work occur (Seto et al., 2012). Urban construction, a point source of pollution (Bu et al., 2014), generates substantial industrial and domestic waste discharges, disrupting the natural link between landscape patterns and bacterial communities. Different landscape patterns alter the input of nutrients or particulate matter into rivers, which provide different nutrients or environmental stresses to bacterial communities, affecting ecological niche processes, and thus influencing bacterial community assembly (Huang and Huang, 2019; Zhao et al., 2021; Shu et al., 2022). Our analysis confirmed significantly higher concentrations of Cl^−^, SO₄^2−^, and NO₃^−^-N in UW compared to AW and NW (Supplementary Table S8). The extensive impervious surfaces and underground drainage networks in urban areas alter surface runoff patterns, resulting in the influx of industrial, agricultural, and domestic wastewater containing pollutants such as organic matter, heavy metals, and nutrients (Kang et al., 2010; Liu et al., 2021). The increased impervious surface area in UW further accelerates the entry of exogenous materials into the waterways (Shu et al., 2020). The influence of landscape patterns on bacterial community structure in UW was less than that in the other two watersheds (*p* > 0.05), which further supports this finding (Figures 6A,D). In addition, the layout of underground pipeline networks in cities makes the relationship between landscape patterns and riverine bacterial communities more complex.

Our results show that the correlations of landscape patterns on bacterial communities were similar at both the near riparian buffer scale (especially the 500 m riparian buffer scale) and the sub-watershed scale across the three watersheds, except that the correlation in the AW was slightly lower at the sub-watershed scale in the dry season. Previous studies have shown that the optimal scale of landscape patterns on bacterial communities in this region is 500 m riparian buffer in the dry season, and it is at 1000 m riparian buffer in the wet season (Shu et al., 2022). The reasons for this inconsistency are possibly due to variations in watershed area, human activities, and the choice of landscape metrics used to represent landscape patterns. Terrestrial landscapes, acting as sources of organic matter, exert both direct and indirect influences on aquatic organisms (Fasching et al., 2020). While some studies highlight the crucial role of riparian buffer zones in intercepting and processing pollutants through runoff (Shi et al., 2017; Hille et al., 2018), other studies emphasize the greater importance of landscape patterns at the sub-watershed scale for regulating water environments due to the broader spatial range of nutrient loading and retention processes. Our results suggest that managing landscape patterns at both the riparian buffer zone (particularly at 500 m) and the sub-watershed scale is equally important for maintaining a healthy riverine bacterial community. Interestingly, the effect of landscape patterns on bacterial communities remained relatively stable across seasons in AW and NW. However, in UW, this influence decreased slightly at the 100 m buffer zone from the dry to the wet season. This shift can be attributed to increased rainfall in the wet season, which introduces more pollutants from urban pipe networks into the river, thereby increasing environmental pressures on bacterial growth and diversity (Johnston and Roberts, 2009; Pokharel et al., 2020). This suggests that the effects of the environmental on river ecosystems need to be considered on a larger scale during the wet season.

### 4.3 Assembly mechanisms of bacterial communities

The results indicated the stronger influence of species sorting, as compared to NW, on bacterial community assembly in the intensive anthropogenic watershed. The dominance of stochastic or deterministic processes in microbial assembly depends on several variables, including nutrient levels (Chase, 2010), environmental stressors (Menéndez-Serra et al., 2023), and spatiotemporal scales (Shi et al., 2018; Fang et al., 2023). Previous research in this region has demonstrated the prevalence of either mass effects (Wang et al., 2019; Zhao et al., 2021) or species sorting (Shu et al., 2022; Wu et al., 2022) as the primary driver of community assembly. Several possible reasons contributed to this phenomenon. Firstly, distinct anthropogenic activities (i.e., urban and agricultural activities) have altered the landscape patterns, water chemistry, and structure of the riverine microbial community (Huang and Huang, 2019). Here, the cities of Xinyu (UW) are rich in iron ore and other mineral resources, which are important industrial areas in Jiangxi Province (Shu et al., 2020). However, residential areas in AW are relatively scattered, with a large number of villages and towns along the river upstream (Supplementary Figure S1). Secondly, the AW exhibited significantly smaller distances in terms of landscape patterns and water chemistry compared to other watersheds in both dry and wet seasons. Surprisingly, we did not observe a distance-decay in AW (Figure 6). Conversely, the NW displayed significantly higher levels of non-freshwater bacteria and ENN_MN (Figure 5; Supplementary Table S4). The non-freshwater populations could have been introduced via runoff or anthropogenic inputs, reflecting the influence of mass effects (Staley et al., 2013; Zhao et al., 2021). These suggest stronger mass effects in NW. Thirdly, spatial distance plays a more important role in explaining planktonic bacterial community structure than environmental factors in large-scale watersheds (He et al., 2021). However, this study focuses on small and medium-sized watersheds in the subtropical region, where the relatively gentle terrain and numerous small reservoirs increase water residence time, thereby enhancing the influence of species sorting (Read et al., 2015; Liu et al., 2018; Zhao et al., 2021).

Our results showed that homogeneous selection and dispersal limitation jointly dominated the bacterial community assembly in the three watersheds. The effect of HoS was AW (71.9%) > UW (42.5%) > NW (33.2%) in the dry season, and UW (53.0%) > AW (47.7%) > NW (37.8%) in the wet season (Figure 8). It is worth noting that the ecological prosses of the bacterial communities in AW (dry season) and UW (wet season) were dominated by deterministic process. HoS is usually considered as a leading factor under the stable state after a disturbance and it may be co-varied with physicochemical variables (Dini-Andreote et al., 2015; Li et al., 2019). In our study, the consistent environmental conditions across the watersheds likely established a stable selective pressure, resulting in HoS as the primary assembly process (Dini-Andreote et al., 2015). Given the relatively small environmental differences among the selected watersheds, HoS emerged as the primary assembly process. Local environmental factors exert a greater influence on bacterial communities than regional factors, as supported by mechanism theories. Here, water chemistry variables such as DOC, NH_4_^+^-N, Cl^−^, and SO_4_^2−^ played crucial roles in shaping the bacterial community structure (Figure 7; Supplementary Tables S5, S6). Variations in dissolved organic matter composition can differentially impact bacterial community distribution by influencing metabolic capabilities (Logue et al., 2016). For instance, *Roseobacter* exhibits high absorption of carbon monomers and amino acids, whereas certain Sphingomonas members thrive in low-carbon environments (Figueroa et al., 2021). Additionally, SO_4_^2−^ and Cl^−^ serve as indicators of anthropogenic pollution sources (e.g., agricultural inputs, sewage leakage, and manure), while essential nutrients such as TP, TN, and NH_4_^−^-N influence the trophic state and eukaryotic microbial structure within aquatic ecosystems (Cai et al., 2022). These findings further support the increased influence of species sorting that was observed in the UW and AW.

## 5 Conclusion

It is important to fully understand the assembly processes of riverine bacterial communities in different anthropogenically disturbed watersheds for the ecosystem health assessments and ecological regulation of rivers. This study revealed that the influence of landscape patterns from riparian buffer zone to sub-watershed on river bacterial community was not significant in the different anthropogenically disturbed watersheds, indicating that the near riparian zone and sub-watershed scale were equally important in protecting the ecological environment of rivers. Homogenous selection leads to a more uniform bacterial community structure and lower diversity, especially in intensive agricultural watersheds. The riverine bacterial communities were mainly affected by changing local environment in intensive agriculture and urban watersheds, rather than exogenous input. These indicated that mass effects and species sorting jointly shaped bacterial community assembly, with species sorting having a greater influence on bacterial community assembly in intensive urban and agricultural watersheds. Our study provides useful and novel insights into the assembly processes of the riverine bacterial community under different anthropogenically disturbed watersheds, and provides a theoretical basis for the ecological management of river environment.

## Data Availability

The datasets presented in this study can be found in online repositories. The names of the repository/repositories and accession number(s) can be found at: https://www.ncbi.nlm.nih.gov/, PRJNA901368.

## Footnotes

^1^https://nasadaacs.eos.nasa.gov/

^2^https://viewer.esa-worldcover.org/worldcover

^3^https://qiime2.org
