# Supplementary material for: Riverine bacterial communities are more shaped by species sorting in intensive urban and agricultural watersheds
Source: Front Microbiol. 2024 Nov 21;15:1463549. doi: 10.3389/fmicb.2024.1463549 (PMC11617543; doi:10.3389/fmicb.2024.1463549)
Supplement: Supplementary file 1 [file Supplementary_file_1.pdf]

Supplementary Materials

Riverine bacterial communities are more shaped by species sorting in intensive urban and agricultural watersheds

Yuanyang She[1,2,3], Peng Wang[1,2*], Jiawei Wen[1,2], Mingjun Ding[1,2], Hua Zhang[1,2], Minghua Nie[1,2], Gaoxiang Huang[1,2]

[1]School of Geography and Environment, Jiangxi Normal University, Nanchang, 330022, Jiangxi, China, [2]Key Laboratory of Poyang Lake Wetland and Watershed Research, Ministry of Education, Jiangxi Normal University, Nanchang, 330022, Jiangxi, China, [3]School of History Culture and Tourism, Longnan Normal University, Longnan 742500, Gansu, China

*** Correspondence:**
Peng Wang

Email: wangpengjlu@jxnu.edu.cn

1 Supplementary Figures

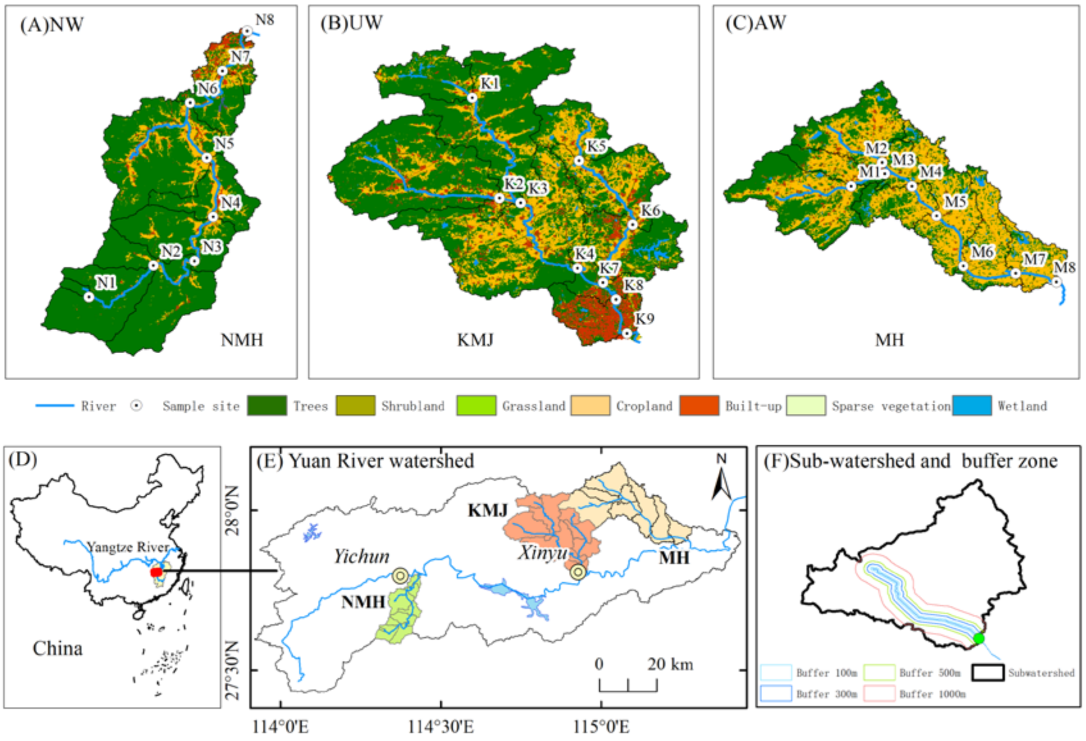

Figure S1. The sampling sites and landuse types in each watershed (NW (A), UW (B), AW (C)), The location of the study area in Yuan River watershed (E), and the riparian buffer zone and sub-watershed (F). NW, natural watershed; UW, intensive urban watershed; AW, intensive agricultural watershed. NMH, Nanmiao River; KMJ, Kongmu River; MH, Meng River.

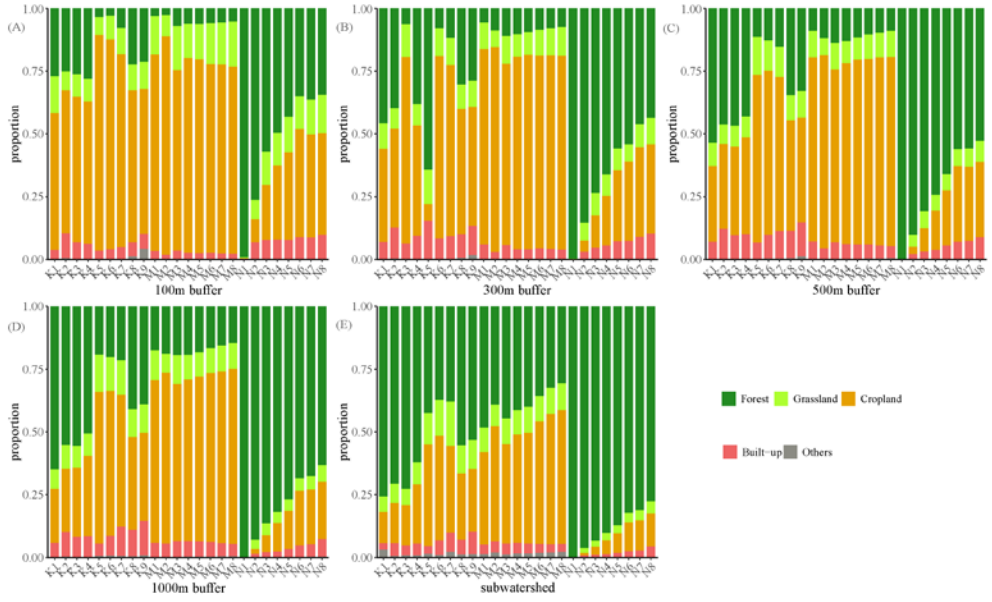

Figure S2. Proportion of Landscape patterns (landuse types) at different scales; (A) 100 m riparian buffer, (B) 300 m riparian buffer, (C) 500 m riparian buffer, (D) 1000 m riparian buffer, (E) sub watershed.

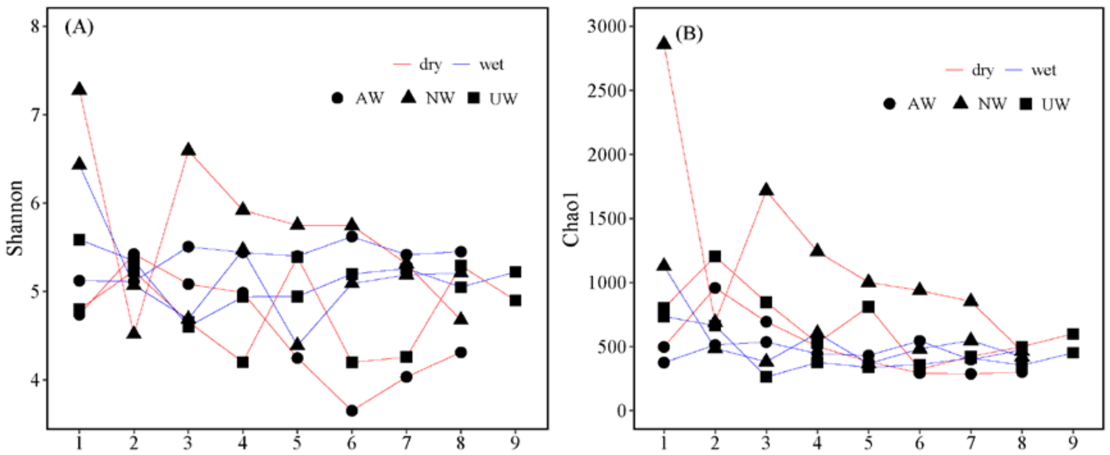

Figure S3. Shannon diversity index (A) and Chao1 richness index (B) in each Sample site (1-9 indicate the number of sampling site, such as K1, N5, M4…). Red line indicated the dry season, blue line indicated the wet season.

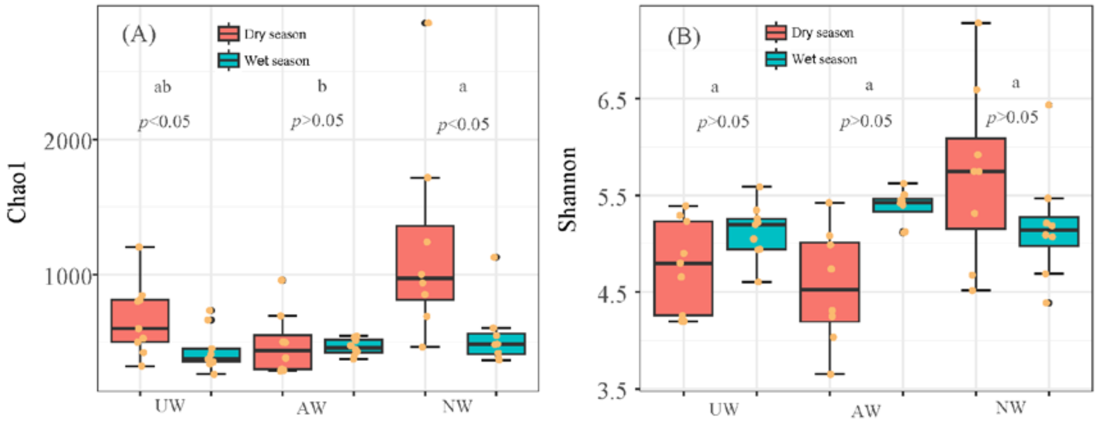

Figure S4. Chao1 richness index (A) and Shannon diversity index (B) in different watersheds, letters a,b represent significant differences of Chao1 and Shannon index in different seasons. Significant differences ($P<0.05$) among dry season and wet season are indicated with different lowercase letters.

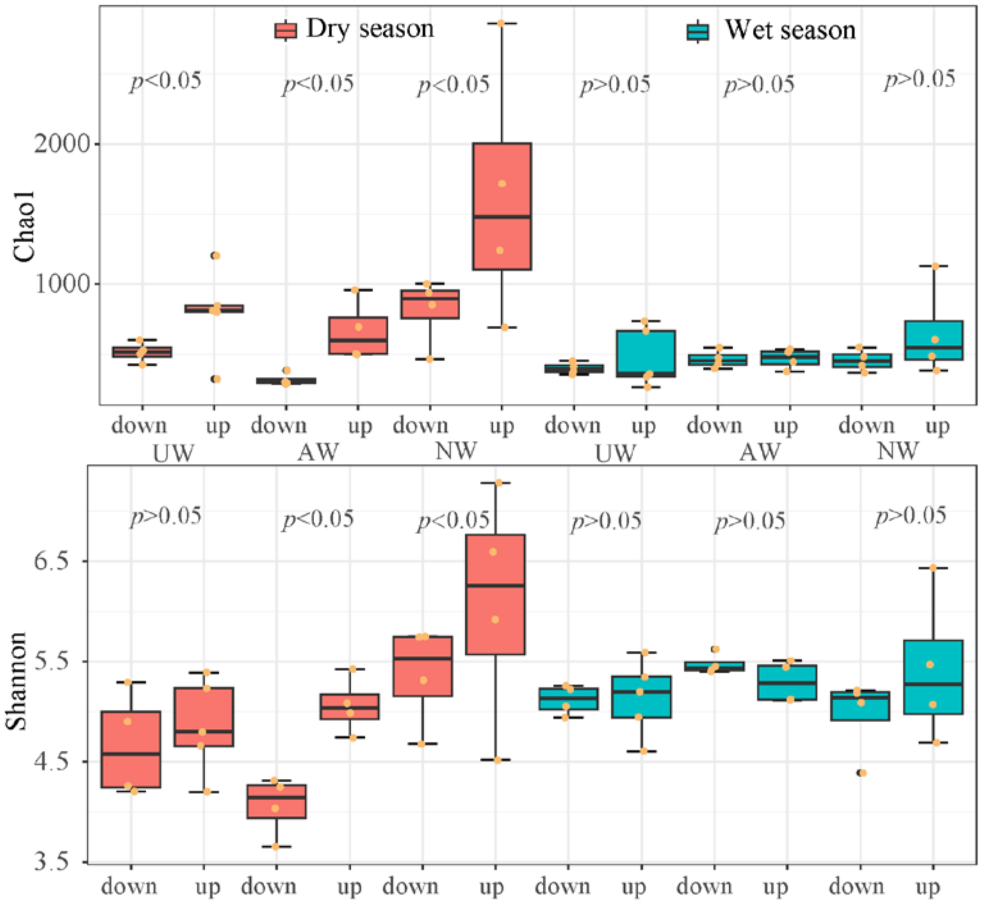

Figure S5. Comparison of Alpha diversity in upstream and downstream rivers in different seasons. Down and up represent the down-stream and upper-stream of a river, respectively. Colors indicate the different seasons.

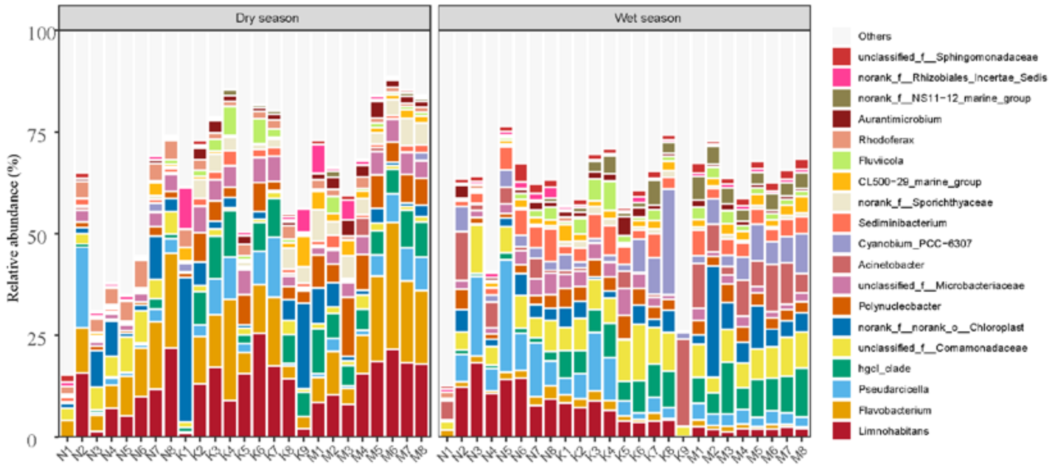

Figure S6. The relative abundances of the dominant genus in the bacterial community in the dry season and the wet season.

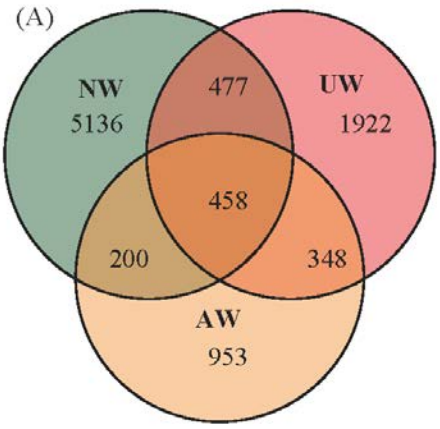

Figure S7. ASV Venn Maps in different seasons and watersheds. (A) and (B) represent the dry season and the wet season, respectively.

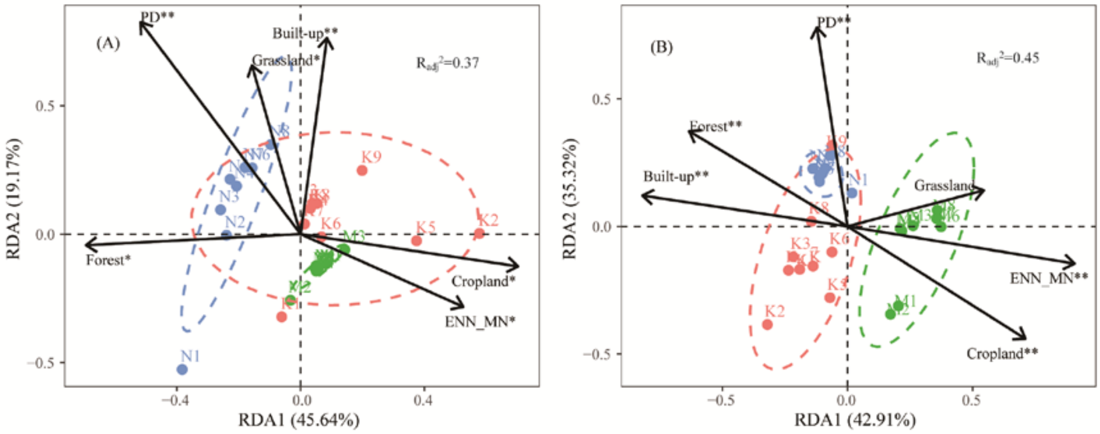

Figure S8. RDA ordinations showing water chemistry parameters variation with landscape pattern types. (A) and (B) represent the dry season and wet season, respectively. ** indicate $P < 0.01$, * indicate $P < 0.05$, and NS. indicate $P > 0.05$.

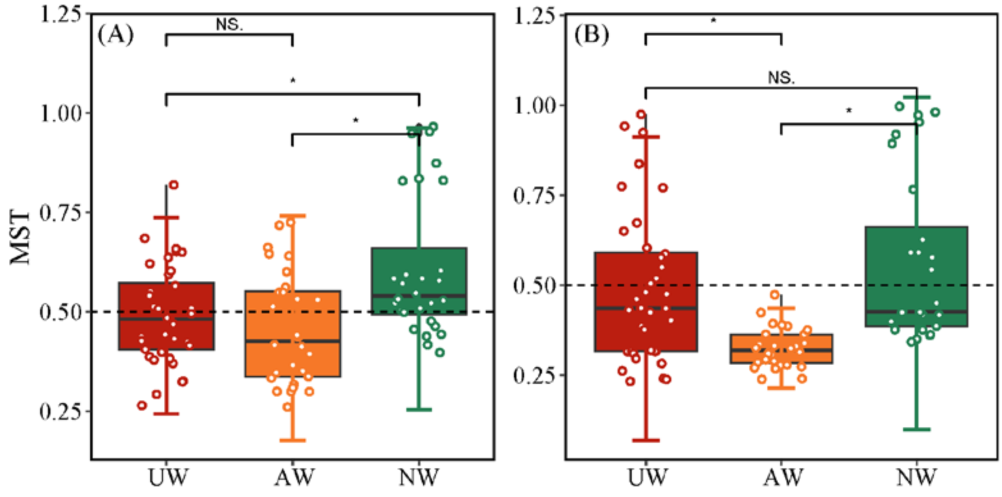

Figure S9. pNST using the null model in the dry season (A) and wet season (B), * indicate $P < 0.05$, NS. indicate $P > 0.05$.

2 Supplementary Tables

Table S1 Proportion of land type composition in each watershed.

	Types	Landuse/%	NW	UW	AW
1	Forest	Trees+Shrub	**77.74**	53.27	30.63
2	Grassland	Grassland+Sparse vegetation	4.79	11.58	10.74
3	Cropland	Cropland	13.15	24.98	**53.29**
4	Built-up	Built-up	4.15	**8.94**	3.26
5	Others	Others	0.17	1.23	2.08

Note: Bold indicates that the land use type has the highest proportion among the three watersheds.

Table S2 Overview of sampling sites.

Watershed	Sampling site	Lon	Lat	watershed area/km^2	Forest /%	Cropland /%	Built-up /%
UW	K1	114.82°E	28.01°N	28.38	81.96	12.43	2.46
	K2	114.85°E	27.92°N	93.69	78.25	16.10	5.09
	K3	114.86°E	27.92°N	212.92	79.18	16.05	4.00
	K4	114.91°E	27.86°N	324.21	70.83	23.66	4.65
	K5	114.91°E	27.95°N	25.96	54.90	40.53	3.15
	K6	114.96°E	27.90°N	69.46	51.49	41.68	5.91
	K7	114.93°E	27.85°N	112.15	55.59	34.61	7.58
	K8	114.94°E	27.84°N	451.36	66.59	26.23	5.96
	K9	114.96°E	27.80°N	479.31	64.77	24.98	8.94
AW	M1	115.03°E	28.02°N	110.34	58.05	36.84	3.82
	M2	115.06°E	28.05°N	82.76	47.63	45.97	4.45
	M3	115.07°E	28.03°N	143.52	54.81	39.70	4.32
	M4	115.10°E	28.02°N	251.54	50.93	43.26	4.30
	M5	115.13°E	27.98°N	294.75	50.32	44.11	3.96
	M6	115.16°E	27.92°N	382.08	45.81	48.81	3.63
	M7	115.23°E	27.91°N	446.62	42.90	51.80	3.36
	M8	115.28°E	27.90°N	488.01	41.33	53.29	3.26
NW	N1	114.33°E	27.62°N	1.53	99.95	0.00	0.04
	N2	114.38°E	27.64°N	28.34	98.27	1.01	0.72
	N3	114.40°E	27.64°N	62.68	95.73	3.24	1.02
	N4	114.42°E	27.67°N	86.93	93.11	5.59	1.28
	N5	114.41°E	27.71°N	104.69	90.45	7.81	1.73
	N6	114.40°E	27.75°N	152.14	86.07	11.47	2.41
	N7	114.42°E	27.77°N	164.24	85.22	11.98	2.61
	N8	114.44°E	27.80°N	175.40	82.50	13.15	4.15

Table S3 The landscape metrics selected in the study.

Landscape metrics	Description	Calculation
Proportion of like adjacencies (PLADJ)	The percentage of adjacent landscape types in the total adjacent landscape (unit: %).	$PLADJ = (\frac{g_{ii}}{\sum_{k=1}^{m} g_{ik}})$
Patch density (PD)	Number of patches per unit area (unit: n/100 ha)	$PD = (n/A) \times 10^6$
Mean Euclidean nearest neighbor index (ENN_MN)	Distance to the nearest neighboring patch of the same land use type based on the edge-to-edge distance (unit: m)	$ENN\_MN = \frac{\sum_{i=1}^{m}\sum_{j=1}^{n} h_{ij}}{n}$

Note: In the equation, p_i is the proportion of the landscape occupied by the land-use type i; p_{ij} is the perimeter of patch j of land-use type i; g_{ik} is the number of adjacencies between pixels of land-use types i and k based on the double-count method; m is the number of patch types present in the landscape; n is the number of patches; h_{ij} is the distance from patch j of land-use type i; a_{ij} is the area of patch j of land-use type i, and A is the total number of cells in the landscape.

Table S4 Mean and standard deviation of Patch density (PD) and Mean Euclidean nearest neighbor index (ENN_MN) in different watershed.

	PD			ENN_MN		
Scale	UW	AW	NW	UW	AW	NW
100	**91.74±17.46**	**80.39±10.73**	**97.07±39.1**	**44.19±4.75**	**50.02±13.01**	46.12±19.76
300	71.81±8.63	62.12±8.34	57.42±28.41	37.72±1.67	42.85±5.12	45.52±20.31
500	68.76±9.03	62.65±6.55	43.43±22.38	36.03±1.87	40.27±4.32	46.16±20.02
1000	61.27±10.63	57.62±2.47	32.63±16.84	38.36±3.22	41.05±1.28	48.06±19.07
Sub-watershed	45.16±9.4	45.7±1.69	19.2±10.55	41.49±2.64	40.06±0.39	**50.98±17.37**

Note: Highest value at different spatial scales within the watershed are shown in bold.

Table S5 Interpretation rate of major environmental variables in different seasons in each watershed based on the CCA model.

Dry season						Wet season					
UW		AW		NW		UW		AW		NW	
variable	*F*	variable	*F*	variable	*F*	variable	*F*	variable	*F*	variable	*F*
**Forest**	**1.37****	**Forest**	**1.95****	**Forest**	**1.62*****	**Forest**	**1.34***	**Forest**	**1.24***	**Forest**	**1.64****
**Grassland**	**1.44*****	**Grassland**	**2.16****	**Grassland**	**1.5****	**Grassland**	**1.46***	**Grassland**	**1.47*****	**Grassland**	**1.52****
Cropland	1.19	**Cropland**	**1.83****	**Cropland**	**1.13***	Cropland	1.27	Cropland	1.14	**Cropland**	**1.14***
Built-up	1.24	**Built-up**	**1.81****	Built-up	1.1.	**Built-up**	**1.71****	**Built-up**	**1.23****	**Built-up**	**1.11***
PD	1.03	**PD**	**1.52****	PD	1.04	PD	1.09	**PD**	**1.2***	PD	0.94
ENN_MN	1.02	ENN_MN	1.22	ENN_MN	1.25*	ENN_MN	1.01	ENN_MN	1.15	ENN_MN	1.23*
**Cl^{-}**	**1.31*****	**SO_4^{2-}**	**1.70****	**Cl^{-}**	**1.69****	**DO**	**1.75*****	SO_4^{2-}	1.08	DOC	1.47
**WT**	**1.19***	**EC**	**1.4***	SO_4^{2-}	1.22.	**WT**	**1.6*****	**WT**	**1.15***	NH_4^+-N	1.06
DOC	1.12	DOC	1.36.	**pH**	**1.15****	**NH_4^+-N**	**1.68****	NH_4^+-N	1.12	Mn	0.95
NH_4^+-N	1.14	**TP**	**1.7****	EC	1.01	Mn	1.15	Mn	0.88	Cu	0.81
Cu	0.97	NH_4^+-N	1.09	Cu	1.12.	**Pb**	**1.46*****	Pb	1.03	Pb	1.05
Fe	0.93	Cu	0.82	Fe	0.91	Fe	1.19	Fe	1	Fe	0.92

Note: * represents P<0.05, **represents P<0.01, ***represents P<0.01. Significant *P* values (<0.05) are shown in bold.

Table S6 Optimal landscape patterns and water chemistry variables obtained for VPA by forward selection.

Season	Watershed	Optimal landscape pattern variables	Optimal water chemistry variables
Dry season	UW	Cropland, Forest, ENN_MN	NH_4^--N, DOC, WT
	AW	Grass, Forest, Built-up	TP, EC, NH_4^--N
	NW	Forest, Built-up, Cropland	Cr, EC, pH
Wet season	UW	Cropland, Forest, ENN_MN, PD	Fe, DO, Mn
	AW	Cropland, Forest, Built-up	NO_3^--N, EC, Fe
	NW	Built-up, Forest, Cropland	WT, Mn, Fe

Table S7 Bacterial biomarkers sensitive in different rivers revealed by LEfSe analysis.

Season	Phylum	Class	Order	Family	Genus	Enriched in the river
Dry season	Actinobacteriota					AW
	Actinobacteriota	*Actinobacteria*				AW
	Actinobacteriota	*Actinobacteria*	*Frankiales*	*Sporichthyaceae*		UW
	Actinobacteriota	*Actinobacteria*	*Frankiales*			UW
	Firmicutes	*Clostridia*				NW
	Pseudomonadota	*Gammaproteobacteria*	*Burkholderiales*	*Burkholderiaceae*	*Polynucleobacter*	AW
	Pseudomonadota	*Gammaproteobacteria*	*Burkholderiales*	*Burkholderiaceae*		AW
	Actinobacteriota	*Actinobacteria*	*Frankiales*	*Sporichthyaceae*	*hgcI_clade*	UW
	Actinobacteriota	*Actinobacteria*	*Frankiales*	*Sporichthyaceae*	*norank_f__Sporichthyaceae*	AW
	Pseudomonadota	*Gammaproteobacteria*	*Burkholderiales*	*Comamonadaceae*	*unclassified_f__Comamonadaceae*	NW
	Firmicutes	*Clostridia*	*Peptostreptococcales-Tissierellales*	*Peptostreptococcaceae*		NW
	Actinobacteriota	*Actinobacteria*	*Micrococcales*	*Microbacteriaceae*	*Aurantimicrobium*	AW
	Firmicutes	*Clostridia*	*Peptostreptococcales-Tissierellales*	*Peptostreptococcaceae*	*Terrisporobacter*	NW
	Firmicutes	*Bacilli*	*Erysipelotrichales*			NW
	Firmicutes	*Bacilli*	*Erysipelotrichales*	*Erysipelotrichaceae*		NW
	Firmicutes	*Bacilli*	*Erysipelotrichales*	*Erysipelotrichaceae*	*Turicibacter*	NW
	Proteobacteria	*Alphaproteobacteria*	*Rhizobiales*	*Rhizobiales_Incertae_Sedis*	*Alsobacter*	AW
	Actinobacteriota	*Actinobacteria*	*Frankiales*	*Sporichthyaceae*	*unclassified_f__Sporichthyaceae*	AW
Wet season	Pseudomonadota	*Gammaproteobacteria*	*Burkholderiales*	*Comamonadaceae*	*Limnohabitans*	NW
	Actinobacteriota	*Actinobacteria*	*Frankiales*	*Sporichthyaceae*		AW
	Actinobacteriota	*Actinobacteria*	*Frankiales*			AW
	Actinobacteriota	*Actinobacteria*	*Frankiales*	*Sporichthyaceae*	*hgcI_clade*	AW
	Bacteroidota	*Bacteroidia*	*Sphingobacteriales*	*NS11-12_marine_group*		AW
	Bacteroidota	*Bacteroidia*	*Sphingobacteriales*	*NS11-12_marine_group*	*norank_NS11-12_marine_group*	AW
	Firmicutes	*Clostridia*				NW
	Patescibacteria					NW

Actinobacteriota	*Actinobacteria*	*Micrococcales*	*Microbacteriaceae*	*Candidatus_Aquiluna*	AW
Bacteroidota	*Bacteroidia*	*Chitinophagales*	*Chitinophagaceae*	*Dinghuibacter*	AW
Firmicutes	*Bacilli*	*Paenibacillales*			UW
Firmicutes	*Bacilli*	*Paenibacillales*	*Paenibacillaceae*		UW
Firmicutes	*Bacilli*	*Paenibacillales*	*Paenibacillaceae*	*Paenibacillus*	UW
Firmicutes	*Clostridia*	*Peptostreptococcales-Tissierellales*			NW
Actinobacteriota	*Actinobacteria*	*Corynebacteriales*			NW
Firmicutes	*Clostridia*	*Peptostreptococcales-Tissierellales*	*Peptostreptococcaceae*		NW
Actinobacteriota	*Actinobacteria*	*Corynebacteriales*	*Nocardiaceae*		NW
Actinobacteriota	*Actinobacteria*	*Corynebacteriales*	*Nocardiaceae*	*Rhodococcus*	NW
Proteobacteria	*Gammaproteobacteria*	*Burkholderiales*	*Methylophilaceae*		UW
Patescibacteria	*Saccharimonadia*				NW
Patescibacteria	*Saccharimonadia*	*Saccharimonadales*			NW
Verrucomicrobiota	*Verrucomicrobiae*	*norank_c__Verrucomicrobiae*			AW
Verrucomicrobiota	*Verrucomicrobiae*	*norank_c__Verrucomicrobiae*	*norank_o__norank_c__Verrucomicrobiae*		AW
Verrucomicrobiota	*Verrucomicrobiae*	*norank_c__Verrucomicrobiae*	*norank_o__norank_c__Verrucomicrobiae*	*norank_f__norank_o__norank*	AW
Proteobacteria	*Gammaproteobacteria*	*Burkholderiales*	*Methylophilaceae*	*norank_f__Methylophilaceae*	AW
Cyanobacteria	*Cyanobacteriia*	*Synechococcales*	*Synechococcaceae*		AW
Cyanobacteria	*Cyanobacteriia*	*Synechococcales*	*Synechococcaceae*	*norank_f__Synechococcaceae*	AW
Verrucomicrobiota	*Verrucomicrobiae*	*Verrucomicrobiales*			AW
Firmicutes	*Clostridia*	*Peptostreptococcales-Tissierellales*	*Peptostreptococcaceae*	*Terrisporobacter*	NW
Proteobacteria	*Gammaproteobacteria*	*Enterobacterales*	*Yersiniaceae*		NW
Patescibacteria	*Parcubacteria*				NW
Proteobacteria	*Gammaproteobacteria*	*Burkholderiales*	*Comamonadaceae*	*Sphaerotilus*	AW
Verrucomicrobiota	*Verrucomicrobiae*	*Verrucomicrobiales*	*Verrucomicrobiaceae*		AW
Bacteroidota	*Bacteroidia*	*Sphingobacteriales*	*Sphingobacteriaceae*		UW

LDA > 3.5, $P < 0.05$.

Table S8 Water chemistry variables concentrations of water samples obtained from the different watersheds.

Seasons	watershed		Cl^- (mg/L)	SO_4^{2-} (mg/L)	pH	DO (mg/L)	EC (μS/cm)	WT (℃)	DOC (mg/L)	TP (mg/L)	NO_3^--N (mg/L)	NH_4^+-N (mg/L)	Mn (μg/L)	Cu (μg/L)	Cd (μg/L)	Pb (μg/L)	Fe (μg/L)
Dry season	UW	Max	45.54	74.15	8.42	12.14	748	11.9	12.68	0.19	2.39	1.38	64.98	1.68	0.05	0.88	486.5
		Min	4.41	21.98	7.74	7.61	354	10.1	4.8	0.01	0.87	0	2.64	0.63	0.02	0.13	224.3
		Mean	**17.31±12.15**	**41.63±15.49**	8.11±0.20	10.32±1.45	**463.33±128.51**	11.01±0.54	**9.12±2.71**	0.07±0.05	**1.28±0.47**	**0.31±0.42**	23.62±18.70	1.18±0.31	**0.03±0.01**	0.35±0.02	**381.58±73.44**
	AW	Max	10.35	24.72	8.38	12.42	368	10.7	10.15	0.74	1.68	0.19	63.2	1.52	0.01	0.73	338.9
		Min	8.5	22.19	7.97	9.85	301	9	7.4	0.05	1.06	0	19	0.99	0.01	0.15	281.6
		Mean	9.44±0.58	23.69±0.76	**8.16±0.13**	11.26±0.88	326.25±20.88	9.83±0.55	8.77±0.95	0.16±0.22	1.27±0.22	0.04±0.07	33.97±13.11	1.18±0.15	0.01±0.00	0.35±0.19	311.66±18.18
	NW	Max	**5.6**	18.67	9.02	13.59	125	12.2	7.4	0.18	1.23	0.37	125.6	1.58	0.02	1.1	195.2
		Min	**1.05**	14.75	7.33	10.91	20.2	7.9	2.37	0.01	0.87	0.03	0.79	0.62	0.01	0.15	21.3
		Mean	3.96±1.37	17.04±1.18	7.93±0.46	**12.13±0.71**	80.44±31.29	10.90±1.21	5.32±1.74	0.10±0.05	0.96±0.11	0.19±0.11	**49.64±39.53**	**1.25±0.28**	0.01±0.00	**0.53±0.26**	123.25±60.16
Wet season	UW	Max	6.54	56.13	8.2	10.34	408	31.2	13.99	0.38	1.08	1.07	80.68	0.94	0.03	2.38	62.62
		Min	2.6	5.85	7.5	5.7	266	25.5	4.32	0.02	0.52	0	13.65	0.65	0.01	0.18	10.83
		Mean	5.08±1.22	**22.37±13.80**	**7.92±0.24**	7.65±1.42	**329.78±41.52**	28.72±1.74	7.17±2.93	0.12 ±0.11	0.79 ±0.18	0.15 ±0.33	35.61 ±20.33	0.79±0.09	0.02±0.01	0.70 ±0.64	39.58 ±16.33
	AW	Max	8.73	10.56	7.8	7.26	275	30.7	11.59	0.23	0.94	0.09	95.14	2.47	0.03	0.28	140.85
		Min	6.09	6.33	7.6	4.73	192.9	28.7	8.34	0.16	0.51	0	14.74	1.07	0.01	0.11	40.3
		Mean	**7.04±0.72**	9.12±3.85	7.67±0.08	6.46±0.72	231.23±31.77	**29.64±0.83**	**10.14±1.78**	**0.22±0.06**	0.76±0.12	0.13±0.33	**45.23±26.05**	**1.72±0.51**	0.01±0.01	0.18±0.06	60.66±37.48
	NW	Max	2.98	3.99	7.9	9.73	90.2	27	9.48	0.24	0.48	1.4	56.74	1.37	0.03	0.62	121.73
		Min	0.67	1.01	7.3	7.22	15.25	19.5	2.9	0.14	0.11	0.18	1.14	0.82	0.01	0.18	9.73
		Mean	1.68±0.78	2.19±0.87	7.65±0.19	**8.74±0.71**	58.14±21.87	24.48±2.12	6.80±2.23	0.19±0.03	0.25±0.10	**0.61±0.44**	27.95±17.72	1.05±0.18	0.02±0.01	0.42±0.17	**78.08±38.53**

Note: Max indicates maximum value; Min indicates minimum value; Mean indicates mean value. Highest value among the watershed in same season are shown in bold.
